# Role of Purine-Rich Regions in Mason-Pfizer Monkey Virus (MPMV) Genomic RNA Packaging and Propagation

**DOI:** 10.3389/fmicb.2020.595410

**Published:** 2020-11-05

**Authors:** Lizna Mohamed Ali, Fathima Nuzra Nagoor Pitchai, Valérie Vivet-Boudou, Akhil Chameettachal, Ayesha Jabeen, Vineeta N. Pillai, Farah Mustafa, Roland Marquet, Tahir A. Rizvi

**Affiliations:** ^1^Department of Microbiology & Immunology, College of Medicine and Health Sciences, United Arab Emirates University, Al Ain, United Arab Emirates; ^2^Architecture et Réactivité de l’ARN, UPR 9002, IBMC, CNRS, Université de Strasbourg, Strasbourg, France; ^3^Department of Biochemistry, College of Medicine and Health Sciences, United Arab Emirates University, Al Ain, United Arab Emirates; ^4^Zayed Center for Health Sciences, United Arab Emirates University, Al Ain, United Arab Emirates

**Keywords:** Mason-Pfizer monkey virus, RNA packaging, RNA secondary structure, single-stranded purines, base paired purines, RNA-Gag interaction, retroviruses, SHAPE

## Abstract

A distinguishing feature of the Mason-Pfizer monkey virus (MPMV) packaging signal RNA secondary structure is a single-stranded purine-rich sequence (ssPurines) in close vicinity to a palindromic stem loop (Pal SL) that functions as MPMV dimerization initiation site (DIS). However, unlike other retroviruses, MPMV contains a partially base-paired repeat sequence of ssPurines (bpPurines) in the adjacent region. Both purine-rich sequences have earlier been proposed to act as potentially redundant Gag binding sites to initiate the process of MPMV genomic RNA (gRNA) packaging. The objective of this study was to investigate the biological significance of ssPurines and bpPurines in MPMV gRNA packaging by systematic mutational and biochemical probing analyses. Deletion of either ssPurines or bpPurines individually had no significant effect on MPMV gRNA packaging, but it was severely compromised when both sequences were deleted simultaneously. Selective 2′ hydroxyl acylation analyzed by primer extension (SHAPE) analysis of the mutant RNAs revealed only mild effects on structure by deletion of either ssPurines or bpPurines, while the structure was dramatically affected by the two simultaneous deletions. This suggests that ssPurines and bpPurines play a redundant role in MPMV gRNA packaging, probably as Gag binding sites to facilitate gRNA capture and encapsidation. Interestingly, the deletion of bpPurines revealed an additional severe defect on RNA propagation that was independent of the presence or absence of ssPurines or the gRNA structure of the region. These findings further suggest that the bpPurines play an additional role in the early steps of MPMV replication cycle that is yet to be identified.

## Introduction

Retroviruses are versatile mobile genetic elements that have historically been used to study the regulation of eukaryotic gene expression (Pachori et al., 2001; Gong et al., 2007; Ly et al., 2007, 2008; Lyon et al., 2008; Yang et al., 2010). Retroviruses are also ideal for the development of vectors for gene therapy (Hu et al., 2011). Vectors based on the Mason-Pfizer monkey virus (MPMV) offer several advantages. For example: (1) they are phylogenetically distinct from human retroviruses, thus avoiding any recombination issues; (2) MPMV can be expressed efficiently in human cells—the single most important criterion for human gene therapy; and (3) many therapeutic genes may need post-transcriptional regulatory elements, such as the MPMV constitutive transport element (CTE) for their efficient cytoplasmic expression (Bray et al., 1994; Rizvi et al., 1996a, b, 1997; Pasquinelli et al., 1997; Zolotukhin et al., 2001).

Specific packaging of the full-length, unspliced gRNA and exclusion of spliced viral and cellular RNAs by the assembling virus particle is a crucial step in the retrovirus life cycle (Lu et al., 2011; Miyazaki et al., 2011; Ali et al., 2016; Comas-Garcia et al., 2016; Kaddis Maldonado and Parent, 2016; Mailler et al., 2016; Dubois et al., 2018). Packaging of retroviral gRNA occurs concomitantly with viral assembly, which takes place either at the plasma membrane (as in the case of human immunodeficiency virus-1; HIV-1) or in the cytoplasm (as in the case of MPMV) followed by their budding from the infected cell (Coffin et al., 1997). The Gag polyprotein plays a key role in gRNA packaging by selectively recognizing gRNA amongst cellular and spliced viral RNAs (Mailler et al., 2016). Retroviral packaging signal RNAs (known as *psi*-ψ) assume higher-order structures and are confined to the first ∼100 to ∼400 nucleotides (nt) of the gRNA that invariably extend into the *gag* gene (Berkhout and van Wamel, 1996; Das et al., 1997; Jewell and Mansky, 2000; Clever et al., 2002; Browning et al., 2003; D’Souza and Summers, 2005; Mustafa et al., 2005; Lever, 2007; Kenyon et al., 2008, 2011; Johnson and Telesnitsky, 2010; Rizvi et al., 2010; Jouvenet et al., 2011; Ali et al., 2016; Comas-Garcia et al., 2016; Kaddis Maldonado and Parent, 2016; Mailler et al., 2016; Dubois et al., 2018). Based on the cross- and co-packaging studies among distinct retroviruses, it has been proposed that structural motifs (rather than their primary sequence) are crucial during gRNA packaging (Embretson and Temin, 1987; Rizvi and Panganiban, 1993; Yang and Temin, 1994; Das et al., 1997; Yin and Hu, 1997; White et al., 1999; Browning et al., 2001; Balvay et al., 2007; Al Dhaheri et al., 2009, Al Shamsi et al., 2011).

**FIGURE 1:**
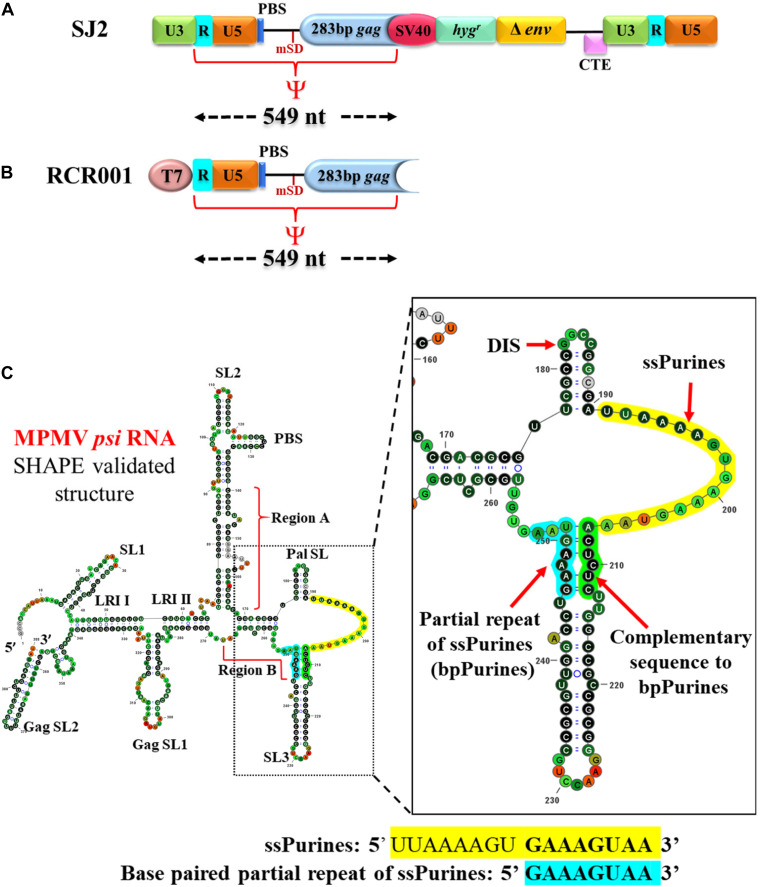
Illustration of the vectors used in the *in vivo* packaging and *in vitro* transcription assays and SHAPE-validated structure of the MPMV packaging signal RNA. **(A)** Schematic representation of the MPMV wild-type sub-genomic transfer vector, SJ2 used in the *in vivo* RNA packaging and propagation assay along with demarcation of boundaries of the RNA packaging determinants denoted as ψ. PBS; primer binding site; CTE; constitutive transport element. **(B)** Schematic representation of the wild-type plasmid used for *in vitro* transcription of the determinants of MPMV RNA packaging. **(C)** SHAPE-validated structure of MPMV RNA packaging determinants. ssPurines, single-stranded purines (yellow); bpPurines, base-paired repeat purines (blue). The green region highlights the sequences complementary to bpPurines. The RNA structure was redrawn in VARNA using SHAPE reactivity data (Aktar et al., 2013).

Mason-Pfizer monkey virus is a *betaretrovirus* that forms intracytoplasmic virus particles unlike other retroviruses, except mouse mammary tumor virus (MMTV; Coffin et al., 1997). Because of the possible use of MPMV-based vectors in human gene therapy, its replicative biology has been investigated extensively with major emphasis on gRNA dimerization, packaging, and RNA propagation (Vile et al., 1992; Harrison et al., 1995; Guesdon et al., 2001; Schmidt et al., 2003; Mustafa et al., 2004; Jaballah et al., 2010; Aktar et al., 2013; Kalloush et al., 2016, 2019; Pitchai et al., 2018). Initial studies on MPMV identified a stretch of sequences at the 5′ end of the viral gRNA important for genome packaging that included sequences from R to the beginning of the *gag* open reading frame, including the major splice donor site (mSD; Figures 1A,B; Harrison et al., 1995; Schmidt et al., 2003). Later, a more systematic mutational analysis of the 5′ end of the MPMV genome revealed that the MPMV packaging determinants were bipartite and resided within two distinct regions: region “A” which included the first 50 nt of the 5′ untranslated region (UTR) and region “B” that encompassed the last 23 nt of the 5′ UTR as well as the first 120 nt of *gag* downstream of mSD (Jaballah et al., 2010). RNA secondary-structure predictions and selective 2′ hydroxyl acylation analyzed by primer extension (SHAPE) revealed that this region folds into a higher-order structure comprising several stable structural motifs (Figure 1C; Jaballah et al., 2010; Aktar et al., 2013). Distinguishing features of the structure included a palindromic stem loop (Pal SL) that was shown to function as the dimerization initiation site (DIS) and U5-Gag long-range interactions (LRIs) were found to be important for maintaining the structure of the entire 5′ leader region (Figure 1C; Aktar et al., 2013; Kalloush et al., 2016). It also revealed a stretch of single-stranded purine-rich sequence (ssPurines) in close proximity of the Pal SL (Jaballah et al., 2010; Aktar et al., 2013). Interestingly, part of the ssPurine-rich sequence was observed to be duplicated as a base-paired sequence at the base of SL3 (named here as “bpPurines”) in region “B” (Figure 1C; Jaballah et al., 2010; Aktar et al., 2013).

The presence of purine-rich sequences in retroviral packaging signal RNA has been proposed to facilitate gRNA packaging by functioning as a potential Gag binding site in HIV-1 (Clever et al., 1995; Abd El-Wahab et al., 2014; Smyth et al., 2015, 2018; Bernacchi et al., 2017), human immunodeficiency virus-2 (HIV-2; Damgaard et al., 1998; Baig et al., 2009), and mouse mammary tumor virus (MMTV; Aktar et al., 2014; Mustafa et al., 2018). The precise role of ssPurines in MPMV life cycle has not been validated, despite indirect evidence for their role in MPMV gRNA packaging (Jaballah et al., 2010). Our previous work suggests that the ssPurines and bpPurines may function in a redundant manner in MPMV gRNA packaging (Jaballah et al., 2010). Therefore, we tested this hypothesis by conducting a methodical mutational analysis of ssPurines and bpPurines complemented by biochemical and structure-function analyses.

## Materials and Methods

### MPMV Strain and Nucleotide Designations

Expression plasmids and MPMV-based vectors were derived from pSHRM15 plasmid (a gift from Dr. Eric Hunter, Emory University, United States) containing sequences of the molecular clone MPMV/6A. The nucleotide numbers refer to the GenBank accession number **M12349** (Sonigo et al., 1986).

### Plasmid Construction

**FIGURE 2:**
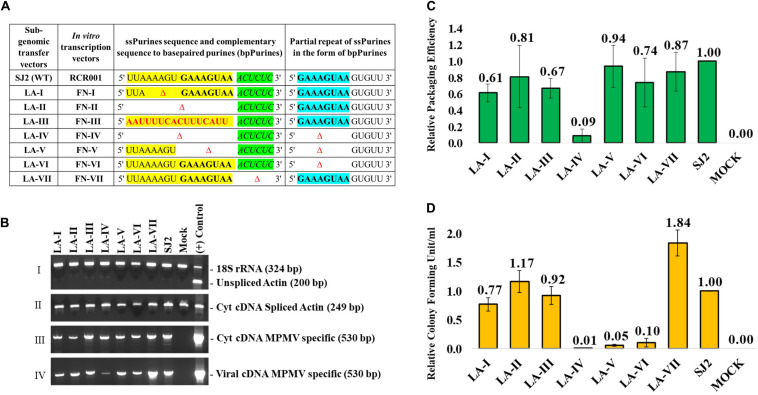
Description of the mutations introduced in ssPurines and bpPurines, RT-qPCR and virus titer analysis of ssPurines and bpPurines mutants to establish their role in MPMV RNA packaging and propagation. **(A)** Table describing the nature of the mutations introduced and cloned both in the context of the sub-genomic transfer and *in vitro* transcription vectors. ssPurines, single-stranded purines (yellow); bpPurines, base-paired repeat purines (blue). The green region highlights the sequences complementary to bpPurines. **(B)** RT-PCR of viral and cytoplasmic cDNA fractions with appropriate controls. **Panel I.** PCR amplification of unspliced β-actin mRNA from cytoplasmic cDNAs. Multiplex amplifications were conducted in the presence of primers/competimer for 18S ribosomal RNA; Genomic DNA (gDNA) used as (+) Control. **Panel II**. PCR amplification of spliced β-actin mRNA from cytoplasmic cDNAs; gDNA used as (+) Control. **Panel III**. PCR amplification of MPMV transfer vector (SJ2)-specific primers from cytoplasmic cDNAs; plasmid DNA for SJ2 used as (+) Control. **Panel IV**. PCR amplification of MPMV transfer vector (SJ2)-specific sequences from cDNAs prepared from the viral RNAs; plasmid DNA for SJ2 used as (+) Control. **(C)** Relative RNA packaging efficiencies for each of the mutant transfer vector RNAs. **(D)** Relative RNA propagation efficiencies for each of the mutant transfer vector RNAs after normalization to secreted alkaline phosphatase (SEAP) expression observed in the transfected cultures and represented as colony forming unit per ml (CFU/ml). The histograms represent data from multiple (to a maximum of five) independent experiments (±SD).

Site-directed mutations were introduced into the *psi* region (from R in the 5′ LTR to 282 nt of *gag*) of the MPMV sub-genomic vector, SJ2 (Jaballah et al., 2010; Figure 1A). SJ2 expresses the *hygromycin B phosphotransferase* gene from an internal simian virus 40 early promoter (SV-Hyg^*r*^) that helps to monitor the effect of mutations introduced into this transfer vector upon propagation of the packaged viral RNA (Figure 1A). Splice overlap extension (SOE) PCR (Gibbs et al., 1994) was employed to introduce the desired mutations. Briefly, two rounds of PCR were conducted using SJ2 as a template to generate intermediate products containing the introduced mutations. Cloning of the mutants was achieved by using an outer sense (S) primer, OTR787 (containing an *Xho*I site at the 5′ end) and an outer anti-sense (AS) primer, OTR788 (containing a *Bam*HI restriction site at the 3′ end) along with internal overlapping primers containing the desired mutations, as previously described (Jaballah et al., 2010; Kalloush et al., 2016, 2019). This resulted in the generation of mutant *psi* fragments containing appropriate flanking cloning restriction sites *(Xho*I at the 5′ end and *Bam*HI at the 3′ end), which were used to clone the fragments containing the mutations into SJ2, resulting in clones LA-I to VII. Details of the oligonucleotides and the templates used for making the mutations are listed in Supplementary Table 1, while the nature of mutations introduced into each clone are described in Figure 2A.

For the *in vitro* dimerization and SHAPE assays, the *psi* region harboring the wild-type and the abovementioned mutations was also cloned into a pUC-based cloning vector, pIC19R (Marsh et al., 1984) to generate the corresponding *in vitro* RNA transcribing clones (Figure 1B). These clones were created by using the corresponding SJ2-based mutants as templates which were amplified using the outer primers, OTR1004 (S) (containing a *Hin*dIII and the T7 RNA polymerase promoter sequence, upstream to the MPMV R sequence) and OTR1005 (AS; containing MPMV *gag* sequences nt 1171-1151 with *Xma*I/*Sma*I site at the 3′ end). The PCR products were cleaved with *Hin*dIII and *Xma*I and ligated to pIC19R that had been previously digested with the same restriction enzymes, resulting in the wild-type (RCR001; Aktar et al., 2013; Kalloush et al., 2016; Figure 1B) and mutant clones capable of *in vitro* transcription from the T7 promoter. *In vitro* RNA transcribing clones containing the mutations were named as FN-I to VII. All the clones were screened by restriction digestion and confirmed by sequencing (refer to Supplementary Table 1 for oligos used in sequencing).

### Cell Culture

Human embryonic kidney cells 293 (HEK 293T) were used for virus production. These cells were maintained at 37°C in the presence of 5% CO_2_ in Dulbecco’s modified Eagle’s medium (DMEM) supplemented with 10% (*v/v*) heat-inactivated fetal bovine serum. To monitor the propagation efficiency of the transfer vector RNAs, the human cervical cancer cell line HeLa T4 was used and maintained at 37°C in the presence of 5% CO_2_ in DMEM supplemented with 7% (*v/v*) heat-inactivated fetal calf serum.

### Transfections and Infections

The SJ2-based mutants were tested *in vivo* to observe the effect of the introduced mutations on gRNA packaging and propagation. Toward this, the MPMV sub-genomic wild-type and mutant transfer vectors that contained the *cis-*acting sequences needed for genome replication, including transcription, polyadenylation, encapsidation, reverse transcription, and integration (Figure 1A), along with the packaging construct, TR301 which expresses Gag-Pol proteins (Browning et al., 2001) and the vesicular stomatitis virus glycoprotein G envelope expression plasmid, MD.G (Naldini et al., 1996), were used in a three-plasmid *trans*-complementation assay, as has been described earlier (Browning et al., 2001; Jaballah et al., 2010; Kalloush et al., 2016, 2019). Transfections of HEK 293T cells with the aforementioned plasmids were carried out using the calcium phosphate transfection method (Invitrogen, United States) according to the manufacturer’s protocol along with pSEAP2-Control vector. The pSEAP2-Control vector expresses secreted alkaline phosphatase (SEAP) and was used to normalize for transfection efficiency of the assay using Great EscAPe SEAP Chemiluminescence kit 2.0 (Clontech, United States), as described previously (Chameettachal et al., 2018; Pitchai et al., 2018; Krishnan et al., 2019). 27 h post-transfection, the pseudotyped virus particles produced from HEK 293T cells were harvested and clarified of cellular debris using low-speed centrifugation, and a portion of it was used to infect HeLaT4 target cells in the presence of 1 μg/ml DEAE dextran, a polycation polymer, to enhance infection efficiency. 48 h post-infection; cells were selected with DMEM medium containing 200 μg/ml of hygromycin B antibiotic (Hyclone, United States) for 10–12 days in order to monitor transfer vector propagation efficiency. The number of resulting hygromycin-resistant colonies obtained per milliliter of the virus supernatant (CFU/ml) is a measure of the propagation efficiency of each mutant transfer vector RNA, which was further normalized to the transfection efficiency using SEAP values. Finally, the resulting normalized CFU/ml was reported relative to the wild-type viral titers. Such *in vivo* packaging and propagation assays allowed us to quantify the effects of the introduced mutations on both RNA packaging as well as its propagation without any uncertainty, since the defective nature of the virions produced limited the assay to a single round of replication (Supplementary Figure 1).

The remaining virus supernatant was clarified of cellular debris by passing through a 0.22-μm cellulose acetate syringe filter and then ultracentrifuged at 70,000 × *g* for 2 h at 4°C on a 20% (*w/v*) sucrose cushion to concentrate the virus particles, as described previously (Jaballah et al., 2010; Kalloush et al., 2016, 2019; Pitchai et al., 2018). The pelleted virus particles were resuspended in TNE buffer [0.25 M Tris (pH 8.0), 0.1 M NaCl, 0.001 M EDTA] and stored in Trizol Reagent (Invitrogen, United States) for virion RNA isolation and subsequently used for real-time quantitative PCR (RT-qPCR).

### RNA Extraction and Real-Time Polymerase Chain Reaction (RT PCR)

HEK 293T cells transfected with different mutant and/or wild-type vectors were harvested from the six-well plates, and a portion of cells were fractionated into nuclear and cytoplasmic fractions, as described previously (Akhlaq et al., 2018; Supplementary Figure 1). The cytoplasmic and packaged viral RNAs were isolated from Trizol following manufacturer’s instructions (Invitrogen, United States). Two micrograms of cellular RNA and the entire virion RNA prep from a six-well plate were subjected to Dnase-treatment (Turbo Dnase, Ambion, United States), followed by PCR amplification with MPMV-vector specific primers OTR1161 and OTR1163 (Supplementary Table 1) to confirm the lack of any carryover plasmid contamination, as described previously (Kalloush et al., 2016; Pitchai et al., 2018). The samples were then converted into cDNAs using M-MLV reverse transcriptase (Promega, United States), and random hexamers, as described earlier (Jaballah et al., 2010; Kalloush et al., 2016). The cDNA samples thus generated were subjected to multiplex PCR using primers specific for unspliced β-actin (OTR582/OTR581; Supplementary Table 1) and 18S ribosomal RNA primer/competimer (QuantumRNA 18S Internal Standards, Ambion) to monitor amplifiability of the cDNAs as well the integrity of the nuclear membrane during the fractionation process. Further PCRs were conducted to analyze spliced β-actin on cytoplasmic cDNAs (OTR582/OTR580; Supplementary Table 1) and MPMV-vector specific primers (OTR1161 and OTR1163) to confirm the expression of cytoplasmic and viral-specific cDNAs.

### Determination of the Relative Packaging Efficiencies of Transfer Vector by Real-Time Quantitative PCR (RT-qPCR)

The RPE of the transfer vector RNAs was determined by conducting RT-qPCR on cDNAs from the cytoplasmic and viral RNA samples. Toward this end, we used a pre-validated MPMV-based custom Taqman gene expression assay developed for this purpose (Kalloush et al., 2016, 2019). Relative quantification of the cytoplasmic and viral packaged RNAs was obtained after normalizing to the endogenous control for which a predesigned human β-actin assay (MGB-FAM) was used, as described previously (Mustafa et al., 2012; Kalloush et al., 2016, 2019). Briefly, equal amounts of cytoplasmic and viral cDNA samples were tested for MPMV and β-actin expression in triplicates for 50 cycles using the QuantStudio^TM^ 7 Flex System (Applied Biosystems, Foster city, CA, United States). The relative quantitation (RQ) values for MPMV expression in the mutants were compared to the wild type to get the relative expression of each mutant vector RNA, both of which were normalized to the β-actin expression levels in each sample. As previously reported, the viral particles were observed to have an equivalent amount of β-actin mRNA irrespective of the amount of viral vector RNA that was packaged, thus providing a good proxy for the amount of virions produced (Mustafa et al., 2012; Supplementary Figure 2). Finally, to determine the packaging efficiency of each vector RNA, the ratio of the viral RQ to the corresponding cytoplasmic RQ for each sample was estimated, and the values represented relative to the wild-type levels.

### Statistical Analysis

The significance of the observed results for the packaging and propagation efficiencies between the wild type and the mutants was established using the standard, paired, two-tailed Student’s *t-*test. A *P-*value of <0.001 was considered to be statistically significant in both the packaging and propagation assays.

### *In silico* Analysis of MPMV RNA Secondary Structure

To establish structure–function relationship of MPMV packaging signal RNA during RNA packaging, the online software Mfold was used to predict the secondary RNA structure for each mutant and wild-type RNA (Mathews et al., 1999; Zuker, 2003). Mfold predicts all optimal and suboptimal RNA secondary structures based on energy matrices by taking into account the minimum free energy of the provided RNA sequence. Next, the predicted structure for each mutant was validated by SHAPE (Merino et al., 2005; Mortimer and Weeks, 2007, 2009; Aktar et al., 2013, 2014; Kalloush et al., 2016, 2019; Mustafa et al., 2018).

### *In vitro* RNA Transcription

The wild-type (RCR001) as well as mutant plasmids created for *in vitro* RNA transcription (FN series of clones; Figure 2A) were linearized with an *Sma*I restriction enzyme located at the 3′ end of the MPMV sequence. The linearized DNA fragments were subjected to *in vitro* transcription using the bacteriophage T7 RNA polymerase (MEGAscript T7 Transcription kit, Thermo Fischer Scientific) as described earlier (Mustafa et al., 2018; Kalloush et al., 2019). A portion of the *in vitro* transcribed RNA was analyzed on 8% acrylamide/8M urea gels to confirm the absence of abortive transcripts, followed by DNase treatment (Turbo DNA, Thermo Fischer Scientific) of the resulting RNA. After phenol/chloroform extraction, the RNAs were further purified on a TSK gel column (TSK Gel G4000SW column, TOSOH Bioscience, Griesheim, Germany) by Fast Protein Liquid Chromatography (FPLC) (AKTA; GE Healthcare Life Sciences, United States) in the presence of a buffer containing 200 mM sodium acetate (pH 6.5) and 1% (*v/v*) methanol. RNA fractions corresponding to the relevant peaks were pooled and ethanol-precipitated. The purified RNA pellets were resuspended in Milli-Q water, quantified by nanodrop (Thermo Fischer Scientific), and 800 ng of each RNA sample was electrophoresed on an 8% acrylamide/8M urea gel to check for its quality.

### Selective 2′ Hydroxyl Acylation Analyzed by Primer Extension (SHAPE) Methodology

The *in vitro* transcribed RNAs were subjected to SHAPE analysis, as described before (Aktar et al., 2013, 2014; Kalloush et al., 2016, 2019; Mustafa et al., 2018). Benzoyl cyanide (BzCN) was used to acylate the 2′-hydroxyl group of the unconstrained nucleotides in the RNA structure, followed by interrogation of each nucleotide using two sets of identical but differentially labeled primers: one set (OTR18 and OTR19) contained the MPMV sequence 5′-AGTTACTGGGACTTTCTCCG-3′ (complementary to MPMV nt 1105-1123) and the second set (OTR22 and OTR23) corresponded to the 5′-CTTACTTTCAGGT CCAACGC-3′ sequence (complementary to MPMV nt 857-875). One primer within each set was labeled with either VIC (OTR18 and OTR22) or NED (OTR19 and OTR23). The NED-labeled primers from each set were used to prepare a ddG sequencing ladder from the untreated RNA samples. The VIC-labeled primers were used for reverse transcription of the modified RNA. The results obtained as electrophoretograms from the capillary electrophoresis of SHAPE-modified RNAs were converted to SHAPE reactivity data by the QuShape algorithm (Karabiber et al., 2013). Reactivity data (Supplementary Table 2) were applied as constraints to the mutant RNA sequence in RNAstructure (version 6) (Reuter and Mathews, 2010) to obtain the validated RNA secondary structure and were redrawn using VARNA software (Darty et al., 2009).

### *In vitro* RNA Dimerization Assays

*In vitro* RNA dimerization was performed on the wild-type (RCR001; Figure 1B) and FN series of mutant clones (Figure 2A) according to the method described previously (Aktar et al., 2013, 2014; Kalloush et al., 2019). Briefly, 300 nM of purified mutant or wild-type RNAs were incubated in dimer (50 mM sodium cacodylate pH 7.5, 300 mM KCl, 5 mM MgCl_2_) or monomer (50 mM sodium cacodylate pH 7.5, 40 mM KCl, 0.1 mM MgCl_2_) buffer for 30 min at 37°C. This was followed by electrophoresis in native 1% agarose TBM (50 mM Tris base, 45 mM boric acid, 0.1 mM MgCl_2_) gel at 4°C. The gels were stained with ethidium bromide and visualized for dimeric or monomeric bands using ultraviolet (UV) transillumination. Band intensities were quantified using ImageLab software (BioImager, Biorad), and the percentage of dimerization was calculated for each RNA employing the following formula: (Intensity of dimer band – Intensity of background)/[(Intensity of monomer band – Intensity of background) + (Intensity of dimer band – Intensity of background)]. Results for each mutant RNA were presented relative to the wild type to determine the effect of mutations on their dimerizing ability.

## Results

### Role of ssPurines in gRNA Packaging and Propagation

To investigate the role of the ssPurines in MPMV gRNA packaging and propagation, we constructed two deletion mutants, LA-I and LA-II along with a substitution mutant LA-III (Figure 2A). LA-I comprised a five-nucleotide deletion (AAAGU) in the former part of ssPurines that is unique to this region as opposed to the latter sequence (GAAAGUAA) which is also repeated as bpPurines at the basal part of SL3 (Figure 1C). This deletion resulted in almost a 50% loss of richness in purines of ssPurines and also a reduction in the size of the ssPurine stretch, while the LA-II mutation created a complete deletion of ssPurines. In LA-III, the ssPurines were substituted with a heterologous non-purine sequence to identify if conserving the sequence or maintaining the secondary structure of the region is vital for viral RNA packaging and propagation. Collectively, these mutants should identify the effect, if any, of the ssPurines region on packaging and propagation of the virus, as well as identify if there were any differential effects between the former and latter purines within the ssPurine region.

These mutants were tested in the biologically relevant *in vivo* packaging and propagation assay which involved co-transfecting the wild-type or mutant transfer vectors mentioned above with the packaging constructs TR301 and MDG (Supplementary Figure 1). The reliability of the *in vivo* RNA packaging assay was ensured by several quality control measures. For example, to ensure the integrity of the nuclear-cytoplasmic fractionation process, end point PCRs specific for unspliced β-actin were performed multiplexed with 18S ribosomal RNA (rRNA) on the cDNAs prepared from the cytoplasmic RNA fractions of the samples (Figure 2B; Panel I). Unspliced β-actin mRNA should be restricted to the nuclear compartment of the cell unless the integrity of the nuclear membrane was compromised during the fractionation process (Tan et al., 1995). Lack of amplification of unspliced β-actin in cDNAs prepared from cytoplasmic RNA fractions not only confirmed the integrity of the fractionation process but also suggested that our samples corresponded to *bona fide* cytoplasmic RNA (Figure 2B; Panel I). Amplifications of 18S ribosomal and spliced β-actin-specific PCRs were conducted in parallel to confirm the presence of amplifiable cDNAs prepared from the cytoplasmic fractions (Figure 2B; Panels I and II, respectively). Next, MPMV transfer vector-specific amplifications were conducted on cDNAs prepared from cytoplasmic fractions as well as from packaged viral RNAs. Such an approach confirmed both efficient nucleocytoplasmic transport as well as stable cytoplasmic expression of the transfer vector RNAs and differential RNA packaging of the mutants (Figure 2B; Panels III and IV, respectively). Finally, having confirmed the integrity of our RNA preparations and the amplifiability of cDNA preparations, we performed real-time quantitative (qPCRs) to assess the amount of packaged RNA in the virions in relation to the RNA transcripts expressed in the cytoplasm of the producer cells, as described previously (Kalloush et al., 2016, 2019). Briefly, qPCRs were conducted for β-actin mRNA packaged into the virus particles, a cellular mRNA which previously has been shown to be packaged passively into the virions at the same levels irrespective of the amount of viral RNA packaged (Mustafa et al., 2012), thus providing an efficient internal control for normalization purposes. Consistently, MPMV particles produced in repeat experiments were observed to have packaged equivalent amounts of β-actin mRNA despite the fact that mutant viral vector RNAs were packaged at different levels, providing an internal control for the amount of virions produced (see dashed boxes in Supplementary Figure 2).

Estimation of the relative packaging efficiency (RPE) of the transfer vector RNAs revealed that despite partial or complete deletion of ssPurines, RNA packaging of the mutant vectors (LA-I, LA-II and LA-III) was not significantly reduced when compared to the wild-type (WT; SJ2) RNA [LA-I (*P* = 0.13); LA-II (*P* = 0.48); LA-III (*P* = 0.04); Figure 2C]. The RNA packaging observation was confirmed by a near identical effect observed on RNA propagation of these mutants, revealing that whatever RNA was packaged by the mutants was propagated successfully to the target cells (compare Figures 2C,D). These results suggest that ssPurines are dispensable for MPMV RNA packaging and propagation. Alternatively, one may also infer that the bpPurines may be compensating for the loss of function of the ssPurines.

### Role of bpPurines in gRNA Packaging and Propagation

To investigate the significance of the redundancy of the purine-rich sequences during MPMV gRNA packaging and propagation processes, two double-deletion mutants involving both the ssPurines and bpPurines were created (LA-IV and LA-V; Figure 2A) along with a mutant with a complete deletion of only the bpPurines (LA-VI; Figure 2A). Mutant LA-IV showed severe defects in RNA packaging (RPE of 0.09; *P* < 0.001) compared to the wild-type SJ2 vector RNA (Figure 2C) which corroborated with a concomitant drastic reduction in the colony-forming units (CFU)/ml observed for this mutant (∼100-fold reduction compared to the WT; *P* < 0.001; Figure 2D). Surprisingly, this was not the case for the double deletion mutant, LA-V and the bpPurine deletion mutant, LA-VI, where the packaging capabilities of these mutant RNAs were nearly similar to that of the wild type [RPE of 0.94 (*P* = 0.67) and 0.74 (*P* = 0.12), respectively, Figure 2C]. However, despite efficient packaging, the propagation of these mutant RNAs (LA-V & VI) were drastically reduced (CFU/ml 0.05 and 0.10, respectively, compared to the WT; *P* < 0.001; Figure 2D). Such an ablated propagation despite efficient RNA packaging is probably because these mutations affected post-packaging events in the viral life cycle such as reverse transcription and/or integration. This assertion is based on the fact that our RNA propagation assay readout is based on successful reverse transcription of the packaged RNA and integration of the reverse transcribed RNA. RNA packaging was drastically affected only by a combined deletion of both the ssPurines and bpPurines sequences (LA-IV; Figure 2C), which could be attributed to structural and/or conformational changes in the RNA of this mutant. Appropriate control experiments, as described above, were conducted to ensure that the wild-type and mutant transfer vector RNAs were expressed efficiently in the cytoplasm and the nucleocytoplasmic fractionation was not compromised in the process (Figure 2B). Considering these controls, effective levels of packaging in both the mutants LA-V and LA-VI suggested that the purine-rich sequences indeed have a redundant role in augmenting gRNA packaging into the assembling virions. Thus, these data demonstrate that ssPurines and its bpPurines function in a compensatory fashion to mediate MPMV gRNA packaging, since their deletions resulted in insignificant effect on RNA packaging of mutants LA-V and LA-VI (Figure 2C).

Finally, mutant LA-VII was designed to study the relevance of maintaining the base pairing in the repeat sequence (bpPurines) at the base of SL3 (Figures 1C, 2A). This mutant carries a deletion of the sequence (5′ ACUCUC 3′) complementary to the base paired region (5′ GAAAGU 3′) in bpPurines; hence, the mutation disrupts base pairing with the repeat region. Interestingly, this mutant did not show any significant defect on gRNA packaging and was encapsidated to nearly the wild-type levels [RPE of 0.87 (*P* = 0.44) relative to the WT; Figure 2C]. Such robust RNA packaging was in agreement with the propagation of the packaged RNA (Figure 2D), keeping in mind all the appropriate controls (Figure 2B). Taken together, the data presented here suggest that: (1) the repeat bpPurines are not important for RNA packaging, and (2) base pairing of these repeat Purines (bpPurines) is neither important for MPMV RNA packaging nor RNA propagation. Furthermore, it suggests that the bpPurines play a vital role in viral RNA propagation since its deletion in the mutants LA-V and VI abrogated RNA propagation completely, despite efficient RNA packaging (Figures 2C,D). Next, the structure of these mutants was analyzed to establish structure-function relationship and to determine whether these phenotypes could be explained by changes in the RNA secondary structure of these mutants.

### Structure-Function Analysis of ssPurines Mutants

The predicted Mfold RNA secondary structures for the ssPurines mutants LA-I to III did not show any noticeable structural disruptions due to deletion or substitution when compared to the wild type (Supplementary Figures 3A–D). To experimentally validate the predicted structures, biochemical probing of the *in vitro* transcribed mutant RNAs was performed employing SHAPE, and their structure–function relationship was established to better understand the results obtained using biological assays.

**FIGURE 3:**
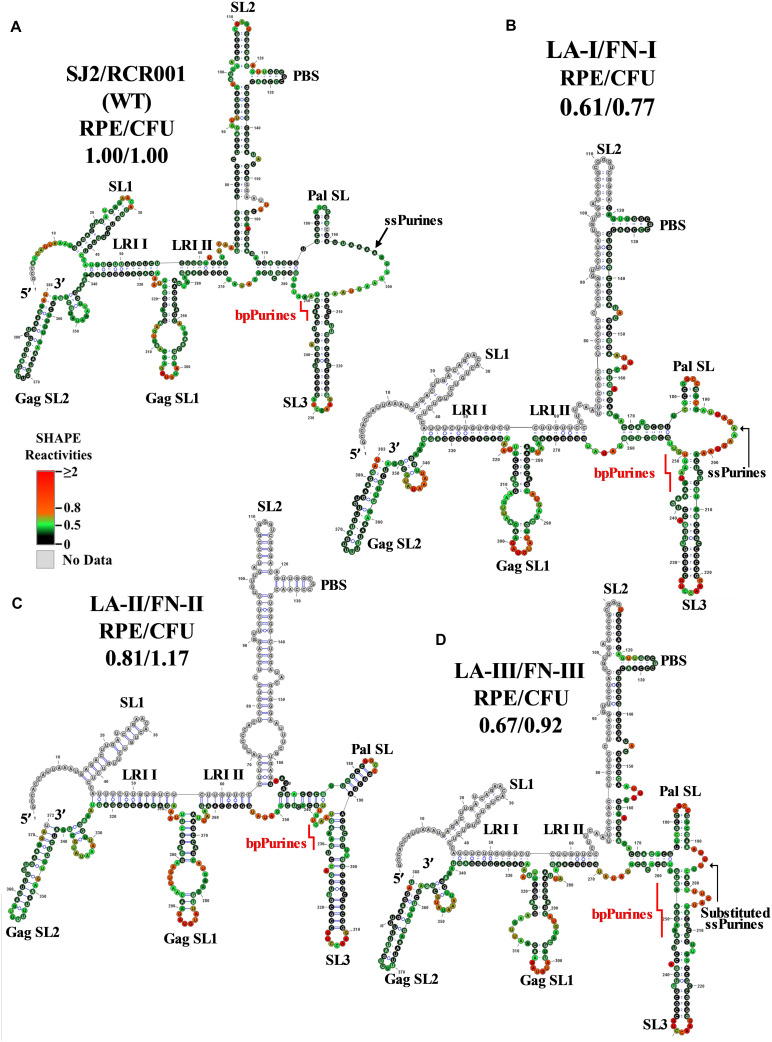
Selective 2′ hydroxyl acylation analyzed by primer extension-validated structures of the wild-type and mutant LA-I/FN-I, LA-II/FN-II, and LA-III/FN-III packaging signal RNAs. **(A)** Wild type (SJ2/RCR001) **(B)** LA-I/FN-I containing deletion of AAAGU (5′ sequence) in ssPurines. **(C)** LA-II/FN-II containing complete deletion of ssPurines. **(D)** LA-III/FN-III containing substitution of ssPurines. The SHAPE reactivities from three independent experiments were averaged and applied to RNAstructure program. The structure with the least minimum free energy was selected and redrawn using VARNA software and the major structural elements are annotated. Nucleotides are color annotated as per the SHAPE reactivities key shown.

The SHAPE validated RNA secondary structure for mutant LA-I harboring five-nucleotide deletion in the former part of ssPurines (Figure 2A) is depicted in Figure 3B. As predicted by Mfold (Supplementary Figures 3A,B), the SHAPE-validated structures of LA-I and wild-type RNAs were found to be very similar (compare Figures 3A,B). Briefly, the LA-I SHAPE-validated structure maintained all the major stem loops, including the two U5-Gag LRIs (LRI I and LRI II) and Pal SL, and only minor changes were observed in the close vicinity of the 5-nucleotide deletion in the ssPurine region. The Pal SL was shortened by a single base-pair at the base, and this shortening of Pal SL was compensated by increasing in size of the linker between SL2 and Pal SL by one nucleotide (Figure 3B). These subtle changes did not compromise the overall RNA secondary structure and the mutant maintained RNA packaging and propagation to wild-type (SJ2) levels (Figures 2C,D).

The SHAPE-validated structure of the LA-II mutant revealed that deletion of the ssPurines, while inducing local remodeling of the RNA secondary structure, did not affect the major structural motifs that have been shown to be important for MPMV gRNA dimerization and packaging (Figure 3C). Briefly, slight nucleotide rearrangements were observed around the base of the SL2 with a noticeable change at the LRI II shortening it by one nucleotide at the U5 region (Figure 3C). Thus, similar to LA-I, the SHAPE-validated structure of LA-II also lends credence to the observation that ssPurines are not crucial for MPMV gRNA packaging process since nearly wild-type levels of RNA packaging and propagation were observed for this mutant (Figures 2C,D). Like LA-I and LA-II, the SHAPE-validated structure of mutant LA-III (substitution of ssPurines with a heterologous non-purine sequence) also revealed conservation of the major structural motifs of the region except for minor changes at the substituted region (only 5 nucleotides remained single-stranded as opposed to 16 in the wild-type structure; Figure 3D vs. Figure 3A). In addition, substitution of ssPurines with a heterologous non-purine sequence in this mutant somehow contributed to a slightly longer stem in both the Pal SL and SL3 (Figure 3D). Corroborating with the SHAPE-validated structure (maintaining all structural motifs needed for RNA packaging and dimerization), this mutant also did not reveal any significant effect on either RNA packaging or propagation (Figures 2C,D). Taken together, analysis of the SHAPE-validated structures of these mutants (LA-I to III) revealed no deleterious changes to the overall RNA secondary structures, and indicated that neither the length of the Pal SL and SL3 stems nor the length or sequence of the ssPurines between these two helices play a significant role in MPMV gRNA packaging.

### Structure-Function Analysis of bpPurines Mutants

**FIGURE 4:**
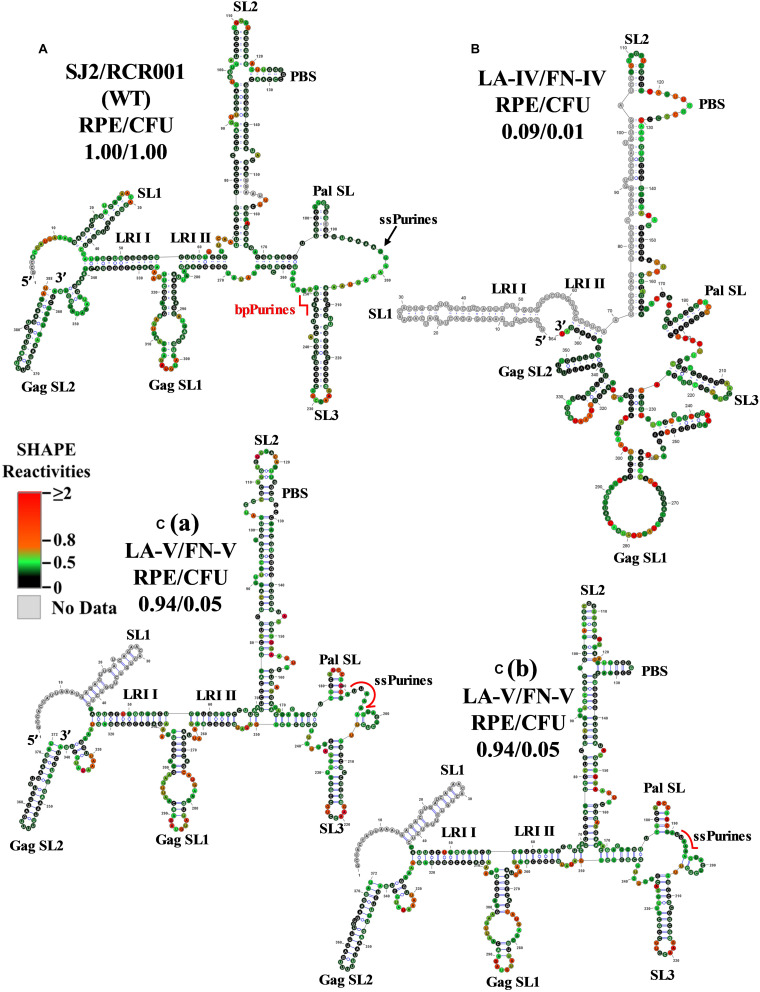
Selective 2′ hydroxyl acylation analyzed by primer extension-validated structures of the wild-type and mutant LA-IV/FN-IV and LA-V/FN-V packaging signal RNAs. **(A)** Wild-type (SJ2/RCR001) **(B)** LA-IV/FN-IV containing deletion of both ssPurines and bpPurines. **[C(a)]** Structure number 1 of the mutant LA-V/FN-V containing deletion of 3′ sequence (GAAAGUAA) of the ssPurines and a complete deletion of bpPurines. **[C(b)]** Structure number 2 of the mutant LA-V/FN-V. The SHAPE reactivities from three independent experiments were averaged and applied to RNAstructure program. The structure with the least minimum free energy was selected and redrawn using VARNA software and the major structural elements are marked. Nucleotides are color annotated as per the SHAPE reactivities key shown.

Mutants with deletions in bpPurines (LA-IV to VI) revealed a complete abrogation of RNA propagation (Figure 2D), indicating a potential role of this sequence in virus replication. Mfold structure predictions of these mutant RNAs (Supplementary Figures 3E–G) suggested preservation of overall RNA secondary structure, despite severe effects on propagation. Thus, SHAPE was conducted on the *in vitro* transcribed RNAs of these mutants to identify any structural changes that may have implications for the observed biological results. In contrast with the Mfold prediction, the SHAPE-validated structure of the double deletion mutant LA-IV (ssPurines as well as bpPurines; Figure 2A) revealed a complete architectural distortion of the region (compare Figures 4A,B). Briefly, crucial structural motifs like U5-Gag LRI I and LRI II that have already been established to be important for MPMV gRNA packaging and propagation (Kalloush et al., 2016) were lost (Figure 4B). Considering that LA-IV was defective for both gRNA packaging and propagation, this observation confirms the importance of maintaining the intact higher-order structure of the packaging signal RNA during MPMV replication. It also reinforces the importance of structural validation of mutants by biochemical probing methods rather than just relying on predictions, especially when the biological data do not correlate with the structure predictions.

The SHAPE-validated RNA structure of mutant LA-V, in which the duplicated sequence (GAAAGUAA) in ssPurines and bpPurines had been deleted and maintained the overall structural motifs critical for gRNA packaging. Noticeably, among the RNA conformers for this mutant, two differently base-paired structures for the primer binding site (PBS) were consistent with the SHAPE data (Figures 4Ca,b). While this double deletion resulted in shortening of SL3, the remaining important structural elements (U5-Gag LRIs and Pal SL) were architecturally maintained (Figures 4Ca,b). These intact RNA domains could have contributed to the efficient packaging of this mutant (Figure 2C). The negative impact on RNA propagation of this mutant could be due to the effect of deletions of the duplicated sequences in the ssPurines and the bpPurines.

**FIGURE 5:**
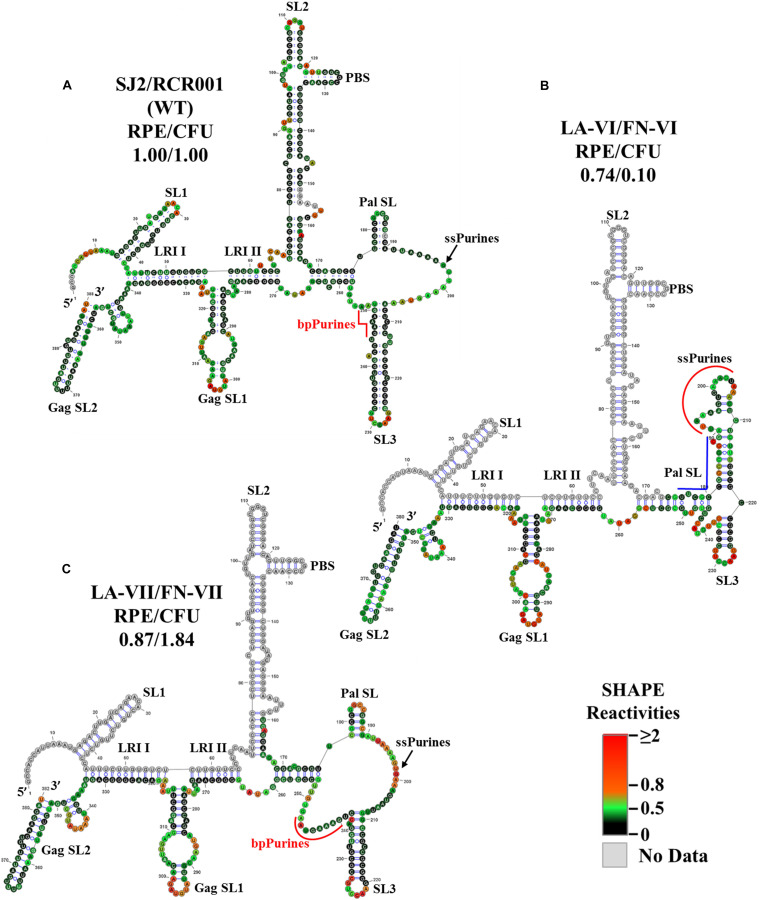
Selective 2′ hydroxyl acylation analyzed by primer extension-validated structures of the wild-type and mutant LA-VI/FN-VI and LA-VII/FN-VII packaging signal RNAs. **(A)** Wild type transfer vector (SJ2/RCR001) **(B)** LA-VI/FN-VI containing complete deletion of bpPurines only. **(C)** LA-VII/FN-VII containing deletion of sequences complementary to bpPurines. The SHAPE reactivities from three independent experiments were averaged and applied to RNAstructure program. The structure with the least minimum free energy was selected and redrawn using VARNA software and the major structural elements are marked. Nucleotides are color annotated as per the SHAPE reactivities key shown.

The SHAPE-validated structure for mutant LA-VI (deletion of only the bpPurines) revealed that while SL1, SL2, GagSL1, GagSL2, LRI-I, LRI-II, and PBS structures were maintained, Pal SL and most of SL3 structure were remodeled (Figure 5B). Thus, conservation of LRI I, LRI II, and of the PBS domain structure might have conferred to the efficient packaging of this mutant (Figure 2C), while loss of the native Pal SL and SL3 structure might have caused the propagation defect (Figure 2D). Alternatively, it is possible that bpPurines play a non-structural role in the early steps of the MPMV life cycle that has yet to be elucidated. Analysis of the ssPurines in the SHAPE structure of LA-VI revealed that they were found partially base paired (Figure 5B). This indicates that ssPurines do not need to be fully single stranded to allow efficient RNA packaging.

The SHAPE-validated structure of LA-VII containing deletion of the sequence (5′ ACUCUC 3′) complementary to the base paired region in bpPurines was not very different from the wild-type structure (Figure 5C), the only differences being slightly shorter Pal SL and SL3 stems, as well as a longer single stranded region immediately downstream of SL3. Based on these structural data, it is not very surprising that the RNA packaging and propagation of this mutant were very efficient (Figures 2C,D). Based on the results from the bpPurines mutants, LA-IV-LA-VII, it is clear that maintaining an intact higher-order structure for the MPMV packaging signal is important for its efficient viral encapsidation. Moreover, the propagation capabilities of the virus were largely influenced by the presence or absence of the bpPurines on an overall mostly intact gRNA structure.

### *In vitro* Dimerization Capability of the Mutant RNAs

**FIGURE 6:**
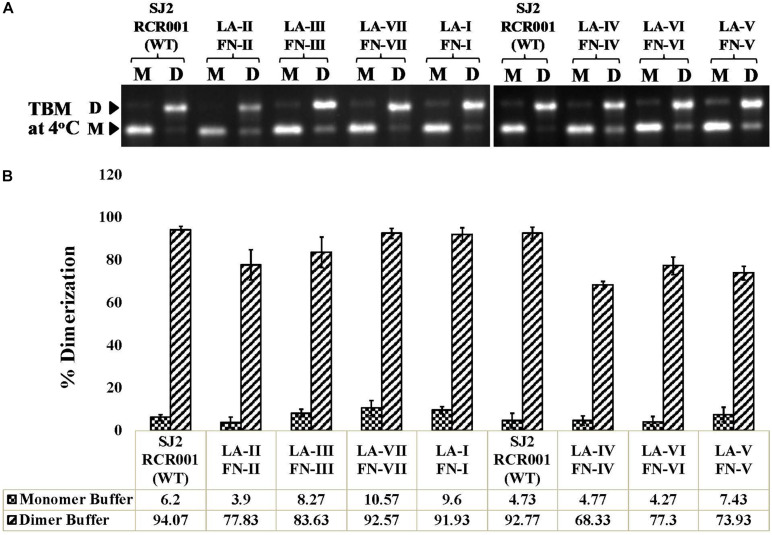
*In vitro* RNA dimerization assay on the wild-type and mutant SHAPE-interrogated packaging signal RNAs. **(A)** Wild-type and mutant RNAs were incubated in monomer (M) or dimer (D) buffer and analyzed by electrophoresis on 1% agarose gel in TBM buffer at 4°C. **(B)** Quantification of the relative ability of each mutant RNA to dimerize compared to the wild-type (SJ2/RCR001) RNA. The experiments were performed in triplicates.

Genomic RNA dimerization and packaging are interconnected events in the retroviral life cycle. Since all the mutants considered in this study showed efficient RNA packaging except for mutant LA-IV, it was important to investigate the dimerization abilities of these mutant RNAs. Thus, the *in vitro* transcribed wild-type (RCR001) and mutant RNAs were incubated in a monomer or dimer buffer and analyzed for the percentage of dimerization after running them on agarose gel in TBM buffer at 4°C. The dimers formed in the TBM buffer at 4°C (Figure 6A) served as a reference for quantifying the percentage of dimerization between the WT and the mutant RNAs (Figure 6B) as described earlier (Aktar et al., 2013, 2014; Kalloush et al., 2016). Interestingly, none of the mutant RNAs tested showed any significant difference in their dimerization potential compared to the wild type (Figure 6). This is consistent with SHAPE-derived structures of mutants LA-I to LA-V and LA-VII since all of them maintained Pal SL, including LA-IV, which has the most dramatically affected secondary structure (Figure 4B). In the case of LA-VI, where the Pal SL was found to be base-paired in the SHAPE-validated structure, the results of dimerization showed no pronounced effect on the dimer forming ability of their respective RNAs (77.3 vs. 92.77 of wild type; Figure 6B). It is conceivable that when the Pal SL is structurally not available, its function could possibly be compensated by palindromic sequence within the PBS region, augmenting RNA dimerization, as has been proposed previously for MPMV and MMTV (Aktar et al., 2013, 2014).

## Discussion

**FIGURE 7:**
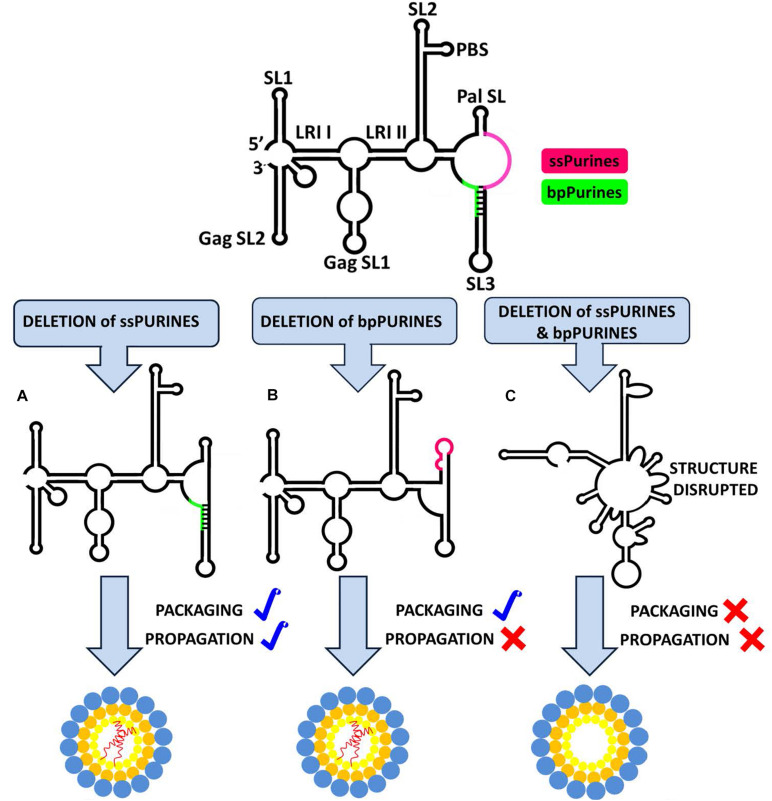
Structural model of MPMV packaging signal RNA containing two purine-rich sequences (ssPurines and bpPurines) which play a redundant role during MPMV gRNA packaging. Single deletion of **(A)** ssPurines or **(B)** bpPurines does not disrupt the overall RNA secondary structure. As a result, RNA is packaged efficiently, suggesting that ssPurines & bpPurines could act as potential Gag binding sites during gRNA packaging. Deletion of bpPurines neither affects RNA structure nor its packaging, but RNA propagation is abrogated. **(C)** Simultaneous deletion of both regions disrupts the overall RNA secondary structure, resulting in abrogation of both RNA packaging and propagation. These data further suggest that in addition to RNA packaging, bpPurines are crucial for some aspects of MPMV replication post-RNA packaging.

The current study was undertaken to establish the role of ssPurines and its base-paired partial repeat, bpPurines, in MPMV gRNA packaging and propagation. The presence of a stretch of purines in the packaging sequences on retroviral gRNA has been proposed to facilitate RNA packaging by functioning as a potential Gag binding site (Paillart et al., 1997; Zeffman et al., 2000; Lever, 2009; Moore and Hu, 2009; Moore et al., 2009; Didierlaurent et al., 2011; Bell and Lever, 2013; Abd El-Wahab et al., 2014; Mustafa et al., 2018). In support of this hypothesis, a purine-rich internal loop in the close proximity of DIS has recently been shown to bind efficiently to purified full-length HIV-1 Gag (Abd El-Wahab et al., 2014; Smyth et al., 2015, 2018; Bernacchi et al., 2017). The SHAPE-validated structure of MPMV packaging signal RNA revealed a stretch of ssPurines (in close proximity of DIS), as well as a partial repeat in the form of bpPurines (Aktar et al., 2013). Therefore, we hypothesized that these stretches of purines could function either at the sequence or structural levels in mediating MPMV gRNA packaging, possibly by functioning as potential Gag binding sites. Based on the data generated while testing this hypothesis, employing mutational, biological, structural, and biochemical analyses allowed us to propose a model shown in Figure 7. According to this model: (1) exclusive deletion of either the ssPurines or bpPurines does not affect MPMV gRNA packaging, (2) deletion of bpPurines, irrespective of the presence or absence of ssPurines affects RNA propagation severely, (3) biochemical probing by SHAPE reveals the structural basis for severe defects in RNA packaging, and finally, (4) RNA dimerization is not affected in any of the mutants. Together, these data confirm that neither ssPurines nor its base-paired duplicated region (bpPurines) serves as unique Pr78^*Gag*^ binding sites on their own; rather, they may act as redundant Pr78^*Gag*^ binding sites for gRNA packaging. These results further suggest that binding of full-length Pr78^*Gag*^ on MPMV gRNA may not be restricted to a single purine-rich domain; hence, additional investigations are warranted to obtain further insights into the Gag binding domain(s) on MPMV gRNA with purified full-length Pr78^*Gag*^ polyprotein.

Experiments performed employing genetic *trans*-complementation assays with the ssPurine mutants (LA-I, LA-II, and LA-III, Figure 2A) did not show any detrimental effect on RNA packaging or propagation (Figures 2C,D). Furthermore, the SHAPE-validated structures for these mutants revealed that the major structural motifs of the MPMV packaging signal RNA, such as the SL1, SL2, and Pal SL (Aktar et al., 2013), and U5-Gag LRIs (Kalloush et al., 2016) that are needed to sustain the overall stability of the RNA secondary structures, were maintained except for minor localized changes due to the introduced mutations (Figures 3B–D). Such a scenario of having localized distortions in the RNA structure not affecting viral replication has been observed earlier, for instance with deletions of the apical part of SL3 that had only a marginal effect on MPMV RNA packaging and propagation (Jaballah et al., 2010; Kalloush et al., 2019). These observations suggest that minor structural changes to the region in and around ssPurines in the MPMV packaging signal RNA are tolerable by the virus; hence, mutations in these regions do not have a profound effect on its packaging or propagation. Taken together, these data suggest that ssPurines by themselves do not play a crucial role in MPMV gRNA packaging and propagation.

Earlier structure prediction analyses of a mutant harboring a deletion of the ssPurines suggested that the stretch of duplicated bpPurines may become single stranded, restoring RNA packaging to almost wild-type levels (Jaballah et al., 2010). However, the SHAPE-validated structure for the replication competent mutant LA-II, containing the precise deletion of ssPurines (Figure 3C) resulted in a structure identical to the wild type, suggesting that the bpPurines may not necessarily be unpaired to allow efficient packaging and propagation of the virus. In agreement with the above observation, the SHAPE-validated structures of mutants containing partial deletion of ssPurines (LA-I) or substitution with heterologous sequence (LA-III) did not reveal any conformational changes leading to unpairing of the bpPurine (Figures 3B,D); yet, these mutants maintained efficient RNA packaging and propagation (Figures 2C,D). Mutants containing deletion of bpPurines in the presence as well as absence of ssPurines (LA-IV, LA-V, and LA-VI) were tested to gain insight toward the importance of the bpPurines during gRNA packaging and propagation. Mutant LA-IV (deletion of both ssPurines and bpPurines) was found to be completely incapable of packaging as well as propagation of its RNA (LA-IV; Figures 2C,D). SHAPE-validated structure of this mutant RNA revealed a complete disruption of the RNA secondary structure including the loss of U5-Gag LRIs that have been shown to be critical for MPMV RNA packaging (Kalloush et al., 2016; Figure 4B) suggesting that the ssPurines and bpPurines may be important in maintaining the spatial organization of the higher-order structure of MPMV packaging signal RNA in a somewhat similar manner to U5-Gag LRIs (Kalloush et al., 2016). In order to destabilize the RNA secondary structure of the packaging signal, the LA-VII mutant was designed by deleting the complementary sequence to the bpPurines on the other side of the stem (Figure 2A). In contrast to our expectations, the LA-VII mutant maintained its RNA secondary structure very similar to the wild type; hence, LA-VII was not only successful in packaging its gRNA into the virus particles, but it also produced infectious virions that could efficiently transduce the target cells (Figures 2C,D).

On the other hand, mutants LA-V (deletion of only the duplicated sequence in ssPurines and bpPurines; Figure 2A) and LA-VI (deletion of only bpPurines; Figure 2A) were found to be defective for RNA propagation, despite their gRNAs being efficiently packaged into the nascent virus particles (Figures 2C,D). SHAPE-validated structures for both these mutants established the presence of majority of the RNA structural domains critical for MPMV gRNA packaging (LA-V; Figures 4Ca,b; LA-VI; Figure 5B), and further asserting that bpPurines by themselves hold no role in facilitating packaging. However, in mutant LA-V RNA, the PBS stem loop revealed two structures in transition with the native wild-type structure among its SHAPE-compatible conformers. In both structures the PBS was base-paired; hence, the lack of propagation could not be attributed to the alternative conformations of the PBS (compare Figures 4Ca,b). In the mutant LA-VI, the Pal SL, ssPurines and the SL3 region were remodeled. Nevertheless, it is noteworthy that all mutant RNAs (LA-I to LA-VII) were successful in forming stable dimers, even in the case of LA-VI where the Pal SL was observed to be base-paired. This could in part be explained if it is assumed that in the absence of Pal SL, dimerization could be initiated by the palindromic sequence in the PBS Pal. Earlier study on MPMV have shown that structural rearrangements at the Pal SL-SL3 region are well accommodated by the virus during its replication process (Kalloush et al., 2019); hence, the loss of propagation of LA-VI packaged RNA could not be related to these observed structural changes. Considering these observations, we speculate that the lack of propagation in these mutants is likely due to the deletion of bpPurines. Therefore, maintaining bpPurines is important for successful MPMV RNA propagation.

In summary, this study suggests that both the ssPurine- and bpPurine-rich sequences play a redundant role in MPMV life cycle perhaps by acting as potential Gag binding sites, while maintaining the overall structure and stability of the MPMV packaging signal RNA. Additionally, our results also suggest a potential role of the bpPurines in the early steps of the MPMV life cycle that affect propagation of the packaged RNA (Figure 7). Further RNA binding studies with purified full-length Gag polyprotein should provide further insights into the nature and context of Gag binding domains necessary for efficient MPMV gRNA packaging. In addition, it would also be interesting to perform *in vivo* or *in virio* probing experiments, as has been done for HIV-1 (Paillart et al., 2004; Wilkinson et al., 2008). Nevertheless, in the case of HIV-1, *in virio* SHAPE data show minimal differences with *in vitro* data, supporting the usefulness of the *in vitro* data.

## Data Availability Statement

All datasets presented in this study are included in the article/Supplementary Material.

## Author Contributions

TR and RM conceived the concept. TR, FM, and RM supervised the project. LA, FP, VV-B, AC, AJ, and VP performed the experiments. LA, FP, AC, FM, RM, and TR contributed toward data analysis. LA and FP wrote the original manuscript. TR, FM, RM, LA, FP, and AC contributed toward reviewing and editing the manuscript. All authors discussed the results and commented on the manuscript.

## Conflict of Interest

The authors declare that the research was conducted in the absence of any commercial or financial relationships that could be construed as a potential conflict of interest.

## Supplementary Material

Supplementary Figure 1 Illustration of the 3-plasmid *in vivo* packaging and propagation assay. **(A)** Graphical representation of the plasmids used to produce pseudotyped virus particles using VSV-G envelope expression vector. **(B)** Schematic depiction of an HEK 293T cells co-transfected with the three plasmids to produce infectious virus particles, which can only replicate to a single round. Unspliced sub-genomic RNA transcribed from the wild-type vector, SJ2, and/or mutant transfer vectors can be packaged into the virions owing to the presence of intact packaging signal, while excluding RNAs transcribed from TR301 and MD.G. **(C)** Transfected cells are fractionated into nuclear and cytoplasmic fractions and analyzed for transfer vector RNA transport and expression. **(D)** Viral particles produced are tested for the amount of RNA packaged by RT-qPCR. Viral supernatants are also used to infect target cells (HeLa T4) to study RNA propagation. After infection, target cells are selected with media containing hygromycin B, allowing only those cells to survive which have been successfully infected since the packaged RNA contains the hygromycin resistance gene. Parts of the figure adapted from Pitchai et al. (2018); Kalloush et al. (2019).

Supplementary Figure 2 β-actin mRNA is packaged into viral particles at the same levels irrespective of the amount of viral RNA present in the virions. **(A)** Amplification plots for packaged viral vector RNAs expressed as ΔRn plotted against cycle numbers (Ct) from a representative experiment. (ΔRn) is the MPMV or β-actin-specific fluorescence signal normalized to the signal for the internal passive control, ROX (Normalized Reporter or Rn) from which the baseline target fluorescence has been subtracted [ΔRn = Normalized Reporter (Rn) – baseline]. The dashed blue boxes show the wide range of Ct values observed for all the mutants and wild-type samples. **(B)** Amplification plots for packaged β-actin mRNA expressed as ΔRn against cycle numbers (Ct) from a representative experiment. The dashed blue boxes show the tight range of Ct values observed for all the mutants and wild-type samples, showing that β-actin mRNA is packaged at the same levels in virions irrespective of the amount of vector RNA packaged. **(C)** Histograms showing the Ct values for β-actin mRNAs observed in all mutants tested. **(D)** Histograms showing the Ct values for β-actin mRNAs observed in all mutants tested relative to the wild type.

Supplementary Figure 3 Mfold structural predictions for the wild-type (SJ2) and mutant packaging signal RNAs. Color coding scheme for major structural motifs: single-stranded purines [(ssPurines) = yellow; base-paired purines (bpPurines) = peacock blue; sequences complementary to (bpPurines) = green; Pal SL (DIS) = red].

Supplementary Table 1 Description of primers and DNA templates used for cloning, sequencing, conventional, and real-time PCR.

Supplementary Table 2 Mean SHAPE reactivity data from triplicate experiments with SD values for wild-type (SJ2/RCR001) and LA/FN clones.
