# Supplementary figures and images for: Role of Purine-Rich Regions in Mason-Pfizer Monkey Virus (MPMV) Genomic RNA Packaging and Propagation
Source: Front Microbiol. 2020 Nov 5;11:595410. doi: 10.3389/fmicb.2020.595410 (PMC7674771; doi:10.3389/fmicb.2020.595410)

# Supplemental Figure 1

**A**

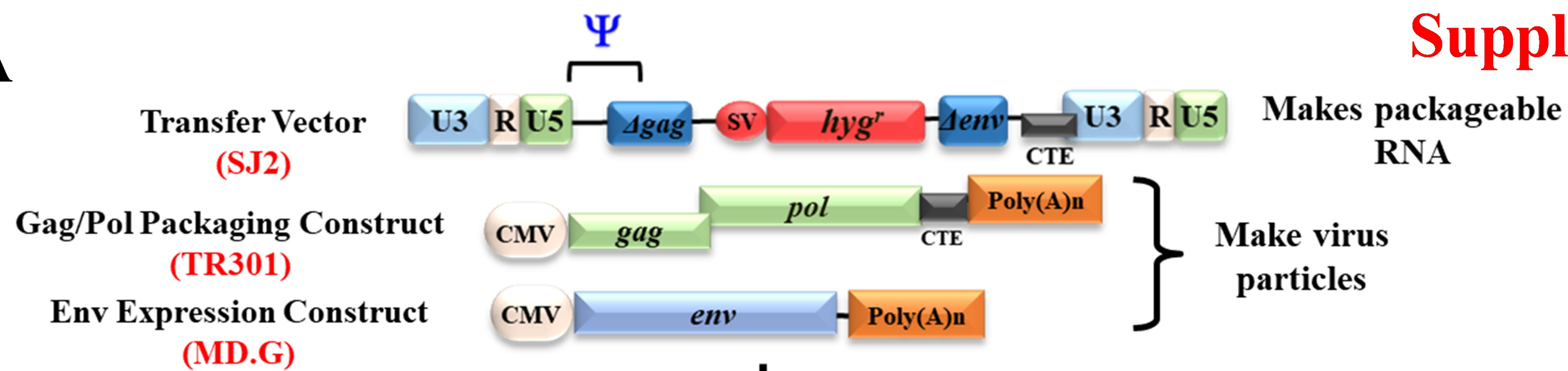

**B**

Transfect producer cells

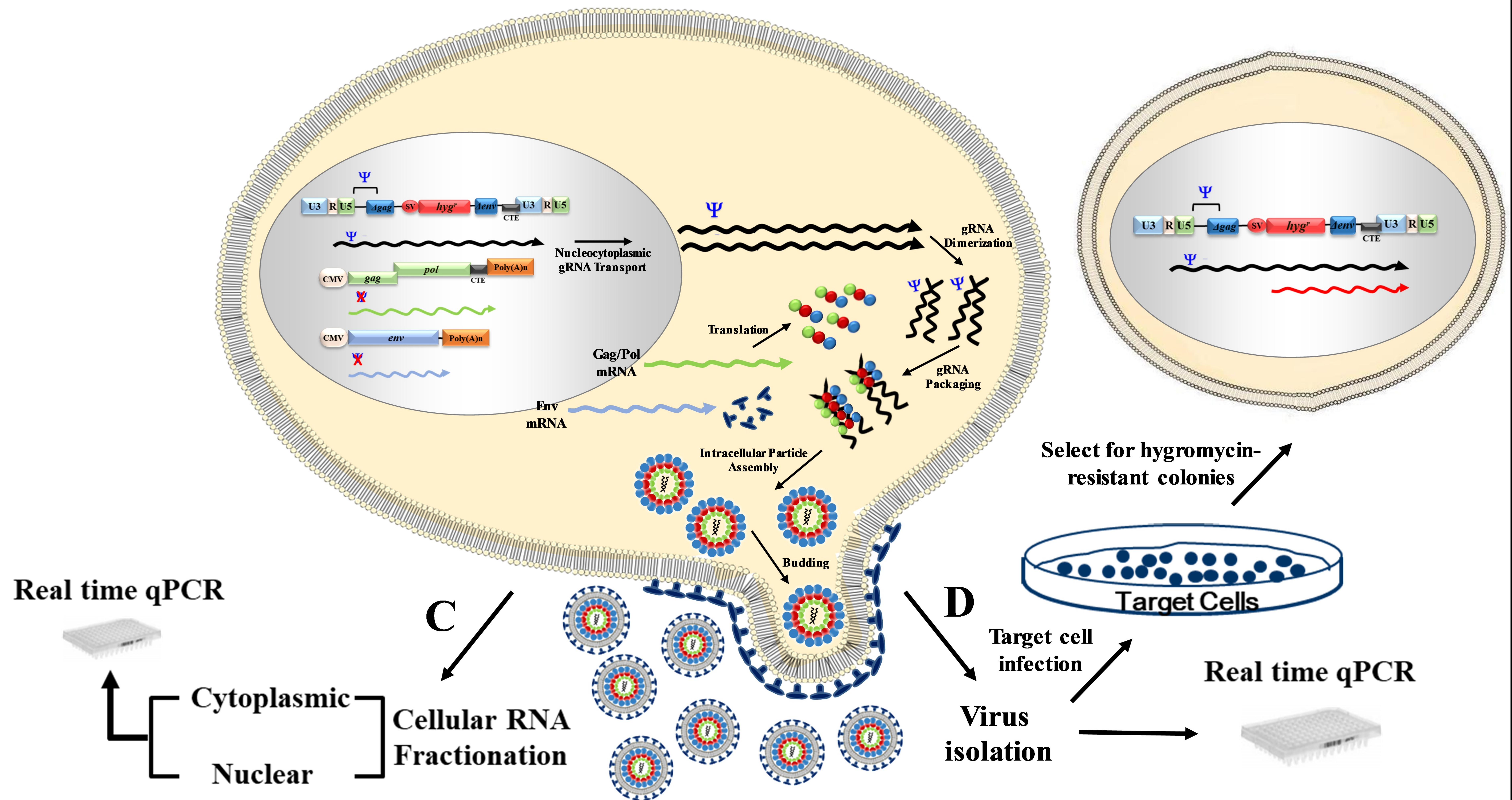

Supplement: Supplementary Figure 1 — Illustration of the 3-plasmid in vivo packaging and propagation assay. (A) Graphical representation of the plasmids used to produce pseudotyped virus particles using VSV-G envelope expression vector. (B) Schematic depiction of an HEK 293T cells co-transfected with the three plasmids to produce infectious virus particles, which can only replicate to a single round. Unspliced sub-genomic RNA transcribed from the wild-type vector, SJ2, and/or mutant transfer vectors can be packaged into the virions owing to the presence of intact packaging signal, while excluding RNAs transcribed from TR301 and MD.G. (C) Transfected cells are fractionated into nuclear and cytoplasmic fractions and analyzed for transfer vector RNA transport and expression. (D) Viral particles produced are tested for the amount of RNA packaged by RT-qPCR. Viral supernatants are also used to infect target cells (HeLa T4) to study RNA propagation. After infection, target cells are selected with media containing hygromycin B, allowing only those cells to survive which have been successfully infected since the packaged RNA contains the hygromycin resistance gene. Parts of the figure adapted from Pitchai et al. (2018); Kalloush et al. (2019). [file Image_1.pdf]

Supplemental Figure 2

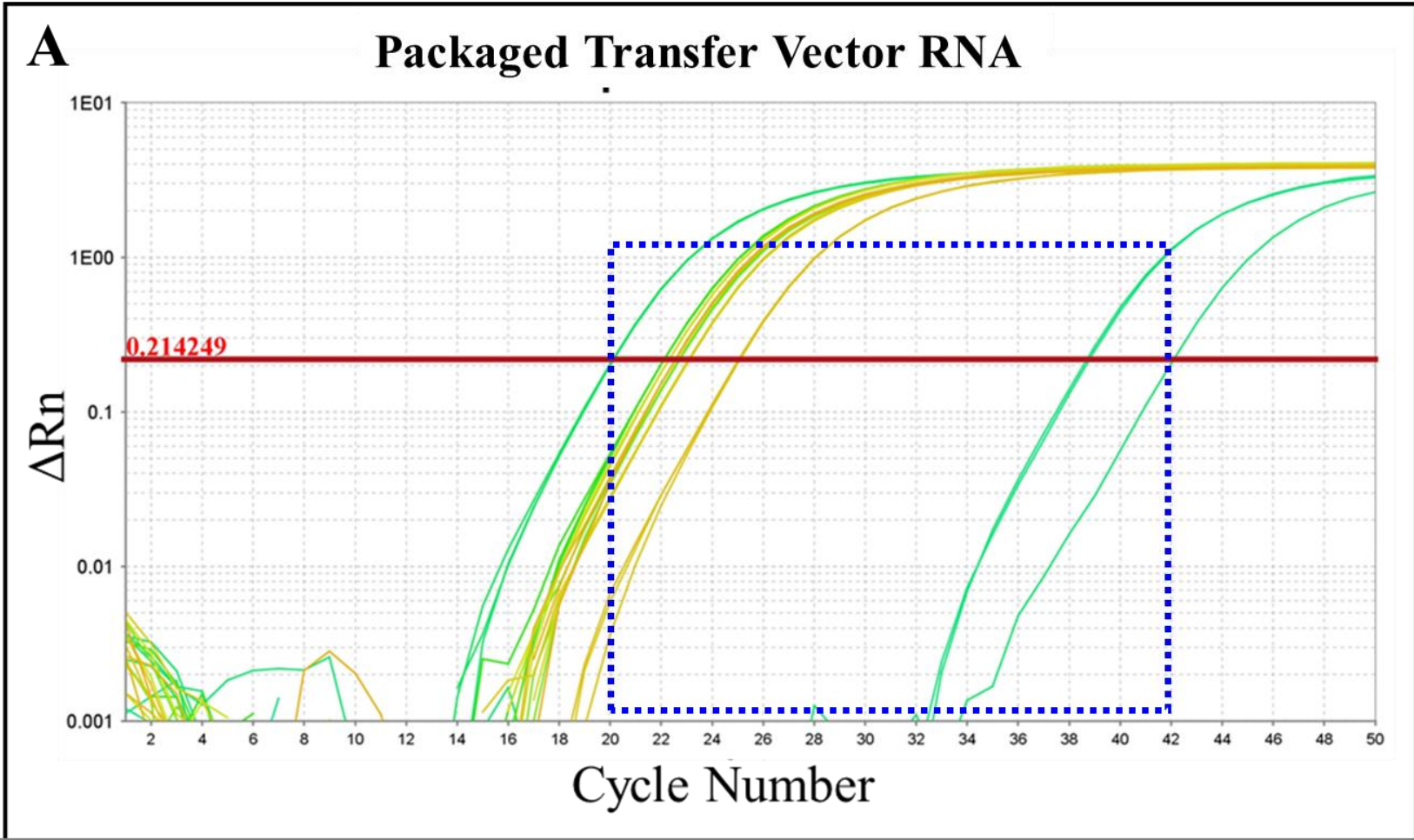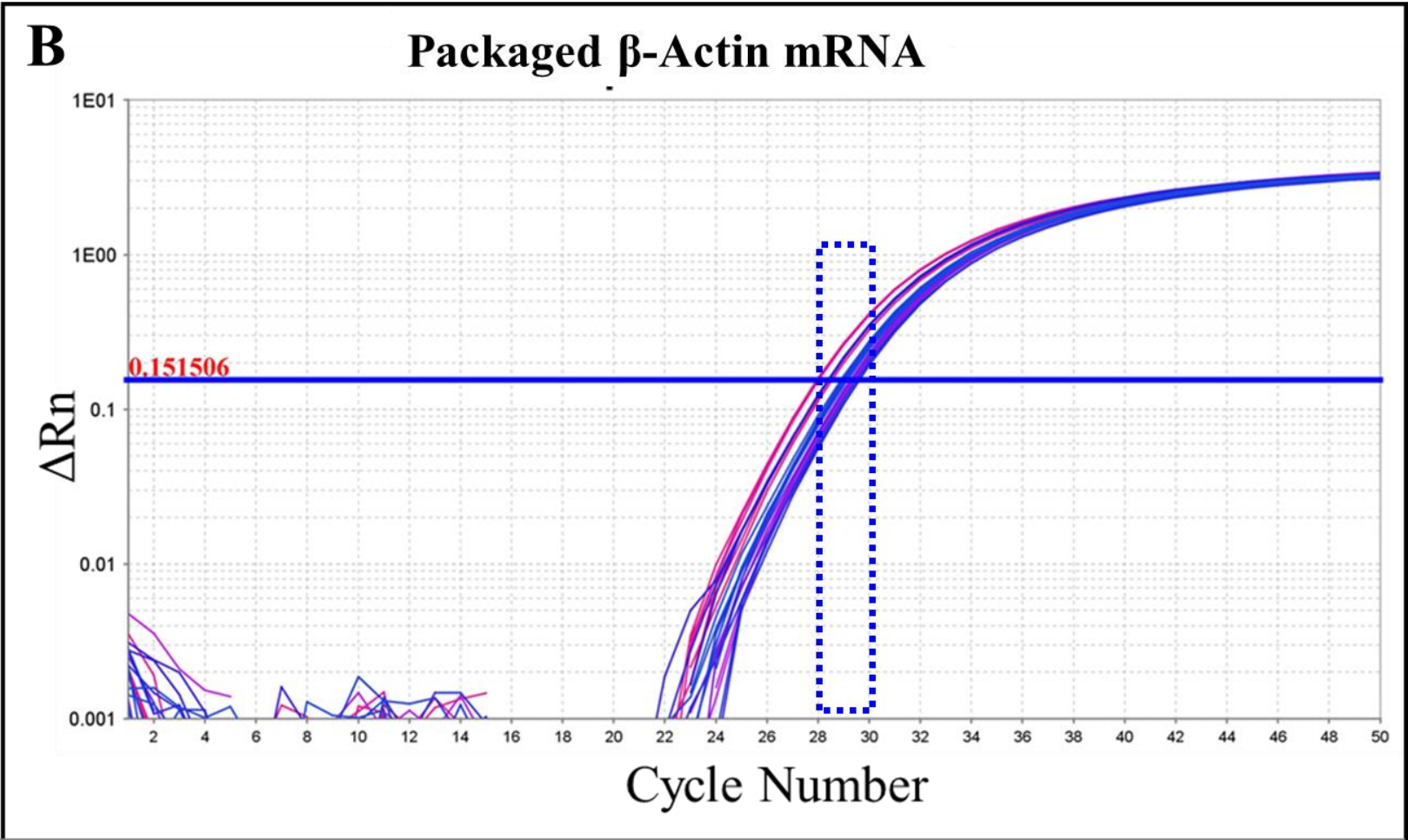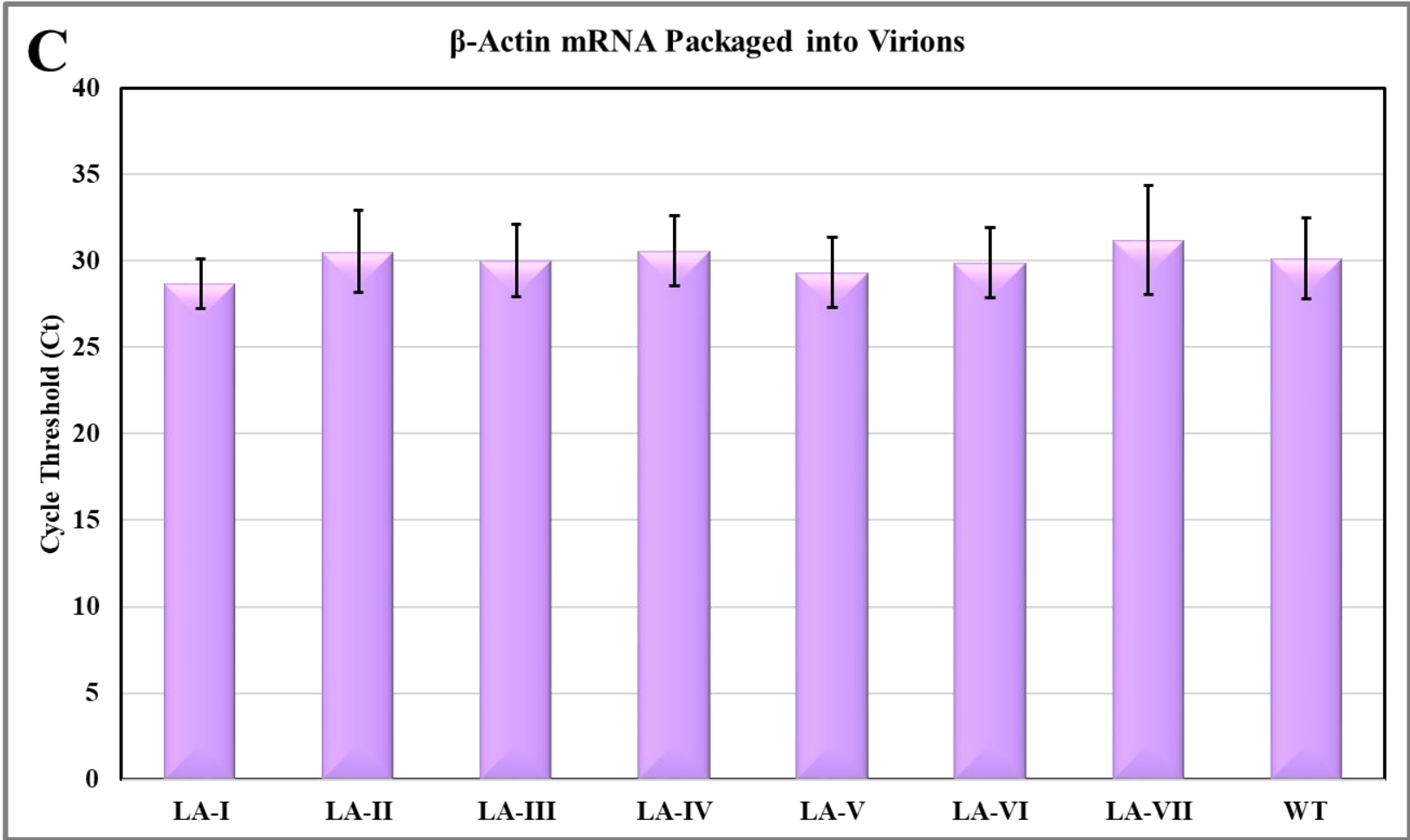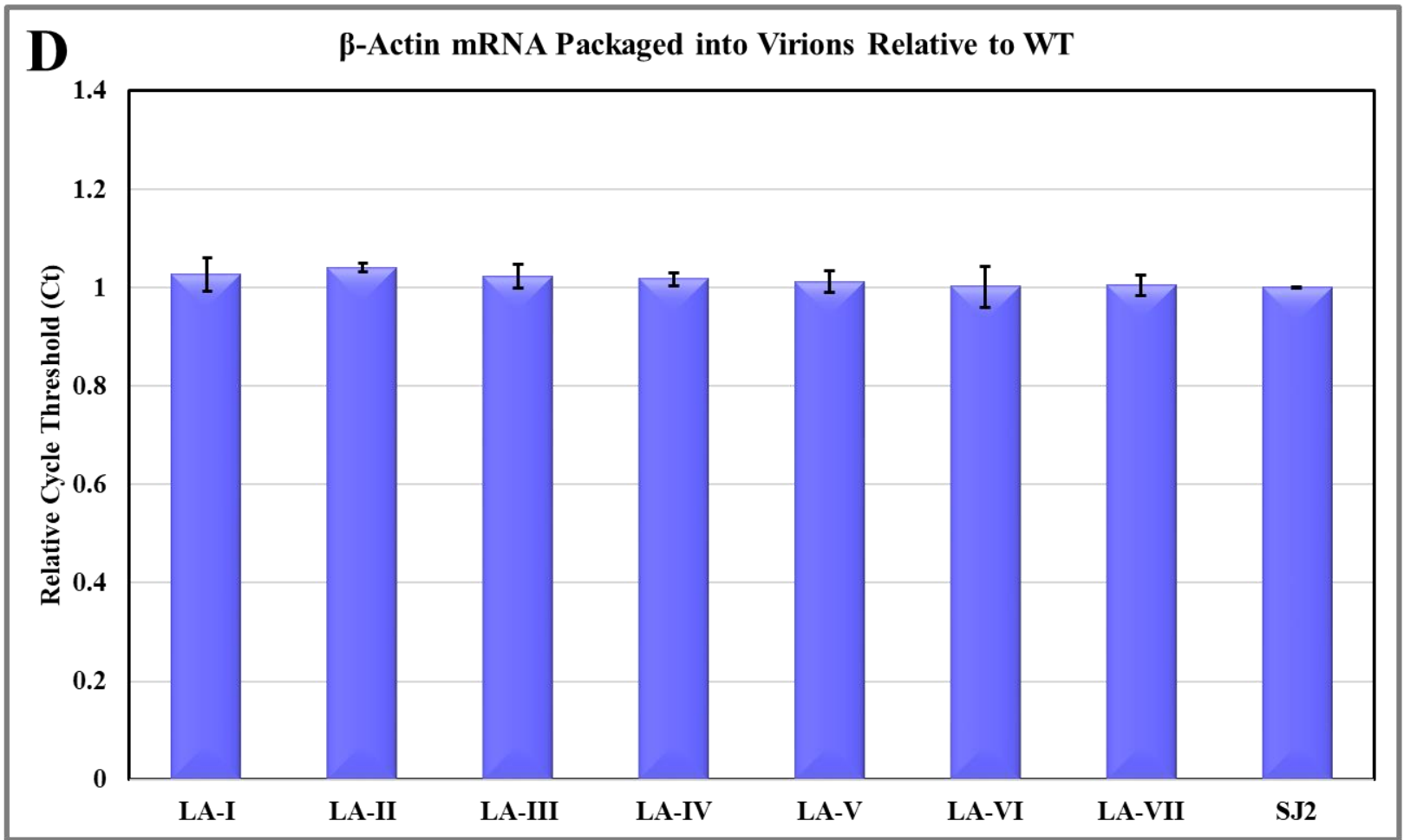

Supplement: Supplementary Figure 2 — β-actin mRNA is packaged into viral particles at the same levels irrespective of the amount of viral RNA present in the virions. (A) Amplification plots for packaged viral vector RNAs expressed as ΔRn plotted against cycle numbers (Ct) from a representative experiment. (ΔRn) is the MPMV or β-actin-specific fluorescence signal normalized to the signal for the internal passive control, ROX (Normalized Reporter or Rn) from which the baseline target fluorescence has been subtracted [ΔRn = Normalized Reporter (Rn) – baseline]. The dashed blue boxes show the wide range of Ct values observed for all the mutants and wild-type samples. (B) Amplification plots for packaged β-actin mRNA expressed as ΔRn against cycle numbers (Ct) from a representative experiment. The dashed blue boxes show the tight range of Ct values observed for all the mutants and wild-type samples, showing that β-actin mRNA is packaged at the same levels in virions irrespective of the amount of vector RNA packaged. (C) Histograms showing the Ct values for β-actin mRNAs observed in all mutants tested. (D) Histograms showing the Ct values for β-actin mRNAs observed in all mutants tested relative to the wild type. [file Image_2.pdf]

# Supplemental Figure 3

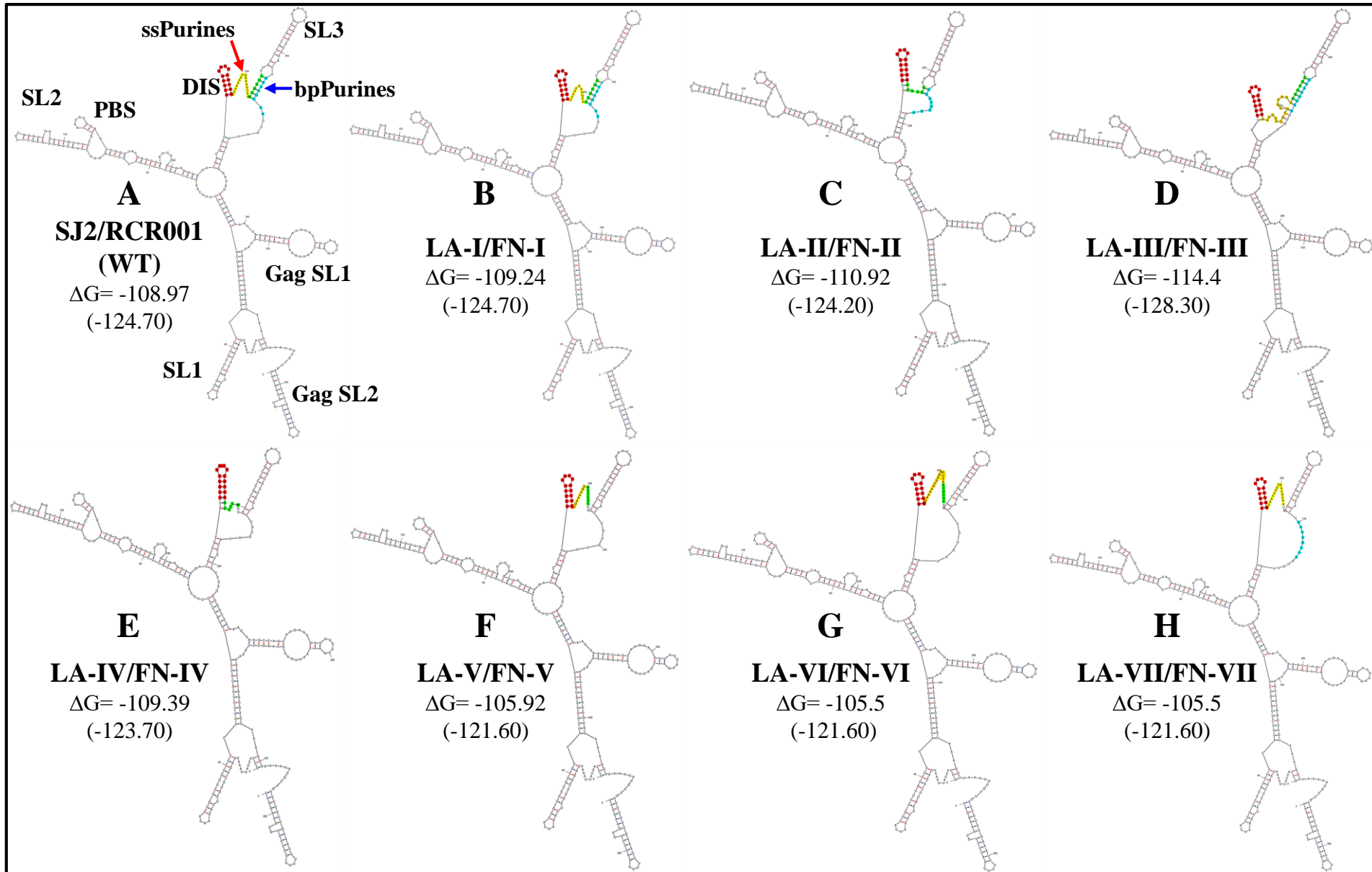

Supplement: Supplementary Figure 3 — Mfold structural predictions for the wild-type (SJ2) and mutant packaging signal RNAs. Color coding scheme for major structural motifs: single-stranded purines [(ssPurines) = yellow; base-paired purines (bpPurines) = peacock blue; sequences complementary to (bpPurines) = green; Pal SL (DIS) = red]. [file Image_3.pdf]
