# Supplementary material for: Role of Purine-Rich Regions in Mason-Pfizer Monkey Virus (MPMV) Genomic RNA Packaging and Propagation
Source: Front Microbiol. 2020 Nov 5;11:595410. doi: 10.3389/fmicb.2020.595410 (PMC7674771; doi:10.3389/fmicb.2020.595410)
Supplement: Supplementary Table 1 — Description of primers and DNA templates used for cloning, sequencing, conventional, and real-time PCR. [file Table_1.pdf]

Supplemental Table 1. Description of primers and DNA template used for cloning, sequencing, conventional, and real time PCR						
**Oligo Name**	***S or AS**	**Clone Names**	**Oligo Sequence**	**DNA template used for SOE PCR**	****Nucleotide (nt) Position and/or Reference**	**Virus Region & Gene**
OTR 787	S	**Outer Primers**	5' CC ctcgagT GTC CGG AGC CGT GCT GCC CG 3'	These oligos were used as outer primers to generate each mutant	2 dummies, *Xho*I, nt 7-21	MPMV 5’ LTR
OTR 788	AS		5' CCC gga tcc TTC TTT CTT ATC TAT CAA TTC TTT AAT TAA G 3'		3 dummies, *BamH*I, MPMV nt 1171-1141	MPMV *gag*
OTR 1348	S	**LA-I**	5' GAA AGT AAA CTC TCT TGG CC 3'	SJ2	MPMV nt 820-839	MPMV U5
OTR 1352	AS		5' GCC AAG AGA GTT TAC TTT CTA ATC GCC GGC CGG CGA ACG 3'		MPMV nt 838-793 with deletion from nt 819-813	MPMV U5
OTR 1012	S	**LA-II**	5' GGC CGG CGA ACT CTC TTG GCC GCC GCG GG 3'	SJ2	MPMV nt 803-847 with deletion from nt 812-827	MPMV U5
OTR 1013	AS		5' GCC AAG AGA GTT CGC CGG CCG GCG AAC GC 3'		MPMV nt 838-794 with deletion from nt 827-812	MPMV U5
OTR 1014	S	**LA-III**	5' AAT TTT CAC TTT CAT TAC TCT CTT GGC CGC CGC GGG 3'	SJ2	Substitution, MPMV nt 828-847	MPMV U5
OTR 1015	AS		5' GCC AAG AGA GTA ATG AAA GTG AAA ATT TCG CCG GCC GGC GAA CGC 3'		MPMV nt 838-794 with substitution from nt827-812	MPMV U5
OTR 1006	S	**LA-IV**	5' GGA CCT GTG TTG CGC TCG GAT ATG GG 3'	LA-2	MPMV nt 861-894 with deletion from nt 867-874	MPMV U5
OTR 1007	AS		5' CAA CAC AGG TCC AAC GCG GCA GGT TC 3'		MPMV nt 880-846 with deletion from nt 874-867	MPMV U5
OTR 1028	S	**LA-V**	5' TTA AAA GTA CTC TCT TGG CCG CCG CGG G 3'	LA-6	MPMV nt 812-847 with deletion from nt 820-827	MPMV U5

OTR 1029	AS		5' GCC AAG AGA GTA CTT TTA ATC GCC GGC CGG C 3'		MPMV nt 838-800 with deletion from nt 827-820	MPMV U5
OTR 1006	S	**LA-VI**	5' GGA CCT GTG TTG CGC TCG GAT ATG GG 3'	SJ2	MPMV nt 861-894 with deletion from nt 867-874	MPMV U5
OTR 1007	AS		5' CAA CAC AGG TCC AAC GCG GCA GGT TC 3'		MPMV nt 880-846 with deletion from nt 874-867	MPMV U5
OTR 1139	S	**LA-VII**	5' GAA AGT AAT TGG CCG CCG CGG GAA C 3'	SJ2	MPMV nt 820-850 with deletion from nt 828-833	MPMV U5
OTR 1140	AS		5' GGC CAA TTA CTT TCA CTT TTA ATC GCC G 3'		MPMV nt 839-806 with deletion from nt 833-828	MPMV U5
OTR 1004	S	**Outer Primers**	5' CCC aag ctt **AAT ACG ACT CAC TAT AGG** GCC ACC ATT AAA TGA GAC TTG ATC 3'	These oligos were used as outer primers to generate each *in vitro* transcribing clones	3 dummies, *Hind*III , T7 promoter sequence, MPMV nt 622-645	MPMV R
OTR 1005	AS		5' AAA ccc ggg TTC TTT CTT ATC TAT CAA TTC 3'		3 dummies, XmaI, MPMV nt 1171-1151	MPMV *gag*
OTR 121	S	**Used for sequencing**	5' GGT TAA ATA TGC TGA TCT TT 3'		MPMV nt 964-983	MPMV *gag*
OTR 198	S		5' ccc gct agc CGC CTA CTC TAC GCC 3'		MPMV nt 1070-1056	MPMV *gag*
OTR 580	S	**β-actin**	5' TGA GCT GCG TGT GGC TCC 3'		*J. Virol.* 69:5607-5620.	Actin Spliced
OTR 581	AS		5' GGC ATG GGG GAG GGC ATA CC 3'		*J. Virol.* 69:5607-5620.	Actin Spliced or unspliced mRNA

OTR 582	S		5' CCA GTG GCT TCC CCA GTG 3'		*J. Virol.* 69:5607-5620.	Actin S-1. Unspliced mRNA
OTR 1161	S	**For PCR following DNase treatment**	5’ GAT CAG AAC ACT GTC TTG TC 3’		MPMV nt 642-661	MPMV R/U5
OTR 1163	AS		5’ CTT TCT TAT CTA TCA ATT CTT TAA 3’		MPMV nt 1169-1146	MPMV *gag*
MPMV_MFAM	-	**qPCR probe**	5' TCGGGACAGTTGGC 3'		MPMV nt 734-747	MPMV U5
MPMV_F	S	**qPCR Forward primer**	5' CTCCTCCAGGTTCCTACTGTTGA 3'		MPMV nt 702-724	MPMV U5
MPMV_R	AS	**qPCR Reverse Primer**	5' TCGTATCCAGCCCCACGTT 3'		MPMV nt 770-752	MPMV U5

Sequence in lower case: non-viral and/or restriction enzyme sequences that were introduced in the oligos for cloning purposes.

Sequence in bold: T7 promoter.

*****S, sense; AS, antisense.

****** The MPMV nucleotide numbering system refers to the genome sequence deposited in the Genbank (accession number M12349) by Sonigo et al. 1986.
