# Supplementary material for: Role of Purine-Rich Regions in Mason-Pfizer Monkey Virus (MPMV) Genomic RNA Packaging and Propagation
Source: Front Microbiol. 2020 Nov 5;11:595410. doi: 10.3389/fmicb.2020.595410 (PMC7674771; doi:10.3389/fmicb.2020.595410)
Supplement: Supplementary Table 2 — Mean SHAPE reactivity data from triplicate experiments with SD values for wild-type (SJ2/RCR001) and LA/FN clones. [file Table_2.pdf]

Supplemental Table 2. **Mean SHAPE reactivity data from triplicate experiments with SD values for wild type (SJ2/RCR001) and LA/FN clones.**

Nucleotide number	Nucleotides	WT/SJ2 (RCR001)	LA-I/FN-I		LA-II/FN-II		LA-III/FN-III		LA-IV/FN-IV		LA-V/FN-V		LA-VI/FN-VI		LA-VII/FN-VII	
		RD*	RD	SD**	RD	SD	RD	SD	RD	SD	RD	SD	RD	SD	RD	SD
1	G	ND	ND	-	ND	-	ND	-	ND	-	ND	-	ND	-	ND	-
2	C	ND	ND	-	ND	-	ND	-	ND	-	ND	-	ND	-	ND	-
3	C	ND	ND	-	ND	-	ND	-	ND	-	ND	-	ND	-	ND	-
4	A	0.24	ND	-	ND	-	ND	-	ND	-	ND	-	ND	-	ND	-
5	C	0.49	ND	-	ND	-	ND	-	ND	-	ND	-	ND	-	ND	-
6	C	0.67	ND	-	ND	-	ND	-	ND	-	ND	-	ND	-	ND	-
7	A	0.64	ND	-	ND	-	ND	-	ND	-	ND	-	ND	-	ND	-
8	U	1.03	ND	-	ND	-	ND	-	ND	-	ND	-	ND	-	ND	-
9	U	0.78	ND	-	ND	-	ND	-	ND	-	ND	-	ND	-	ND	-
10	A	0.70	ND	-	ND	-	ND	-	ND	-	ND	-	ND	-	ND	-
11	A	0.45	ND	-	ND	-	ND	-	ND	-	ND	-	ND	-	ND	-
12	A	0.53	ND	-	ND	-	ND	-	ND	-	ND	-	ND	-	ND	-
13	U	0.38	ND	-	ND	-	ND	-	ND	-	ND	-	ND	-	ND	-
14	G	0.11	ND	-	ND	-	ND	-	ND	-	ND	-	ND	-	ND	-
15	A	0.08	ND	-	ND	-	ND	-	ND	-	ND	-	ND	-	ND	-
16	G	0.10	ND	-	ND	-	ND	-	ND	-	ND	-	ND	-	ND	-
17	A	0.12	ND	-	ND	-	ND	-	ND	-	ND	-	ND	-	ND	-
18	C	0.07	ND	-	ND	-	ND	-	ND	-	ND	-	ND	-	ND	-
19	U	0.12	ND	-	ND	-	ND	-	ND	-	ND	-	ND	-	ND	-
20	U	0.17	ND	-	ND	-	ND	-	ND	-	ND	-	ND	-	ND	-
21	G	0.13	ND	-	ND	-	ND	-	ND	-	ND	-	ND	-	ND	-
22	A	0.46	ND	-	ND	-	ND	-	ND	-	ND	-	ND	-	ND	-
23	U	0.53	ND	-	ND	-	ND	-	ND	-	ND	-	ND	-	ND	-
24	C	0.17	ND	-	ND	-	ND	-	ND	-	ND	-	ND	-	ND	-
25	A	0.31	ND	-	ND	-	ND	-	ND	-	ND	-	ND	-	ND	-
26	G	0.35	ND	-	ND	-	ND	-	ND	-	ND	-	ND	-	ND	-

27	A	0.70	ND	-	ND	-	ND	-	ND	-	ND	-	ND	-	ND	-
28	A	0.89	ND	-	ND	-	ND	-	ND	-	ND	-	ND	-	ND	-
29	C	0.48	ND	-	ND	-	ND	-	ND	-	ND	-	ND	-	ND	-
30	A	0.98	ND	-	ND	-	ND	-	ND	-	ND	-	ND	-	ND	-
31	C	0.19	ND	-	ND	-	ND	-	ND	-	ND	-	ND	-	ND	-
32	U	0.17	ND	-	ND	-	ND	-	ND	-	ND	-	ND	-	ND	-
33	G	0.10	ND	-	ND	-	ND	-	ND	-	ND	-	ND	-	ND	-
34	U	0.05	ND	-	ND	-	ND	-	ND	-	ND	-	ND	-	ND	-
35	C	0.06	ND	-	ND	-	ND	-	ND	-	ND	-	ND	-	ND	-
36	U	0.05	ND	-	ND	-	ND	-	ND	-	ND	-	ND	-	ND	-
37	U	0.08	ND	-	ND	-	ND	-	ND	-	ND	-	ND	-	ND	-
38	G	0.15	ND	-	ND	-	ND	-	ND	-	ND	-	ND	-	ND	-
39	U	0.08	ND	-	ND	-	ND	-	ND	-	ND	-	ND	-	ND	-
40	C	0.06	ND	-	ND	-	ND	-	ND	-	ND	-	ND	-	ND	-
41	U	0.10	ND	-	ND	-	ND	-	ND	-	ND	-	ND	-	ND	-
42	C	0.53	ND	-	ND	-	ND	-	ND	-	ND	-	ND	-	ND	-
43	C	0.45	ND	-	ND	-	ND	-	ND	-	0.30	0.42	ND	-	ND	-
44	A	0.49	ND	-	ND	-	ND	-	ND	-	0.07	0.10	ND	-	ND	-
45	U	0.46	ND	-	ND	-	ND	-	ND	-	0.22	0.28	ND	-	ND	-
46	U	0.33	ND	-	ND	-	ND	-	ND	-	0.19	0.19	ND	-	ND	-
47	U	0.24	ND	-	ND	-	ND	-	ND	-	0.01	0.01	ND	-	ND	-
48	C	0.07	ND	-	ND	-	ND	-	ND	-	0.01	0.01	ND	-	ND	-
49	U	0.24	ND	-	ND	-	ND	-	ND	-	1.47	2.07	ND	-	ND	-
50	U	0.25	ND	-	ND	-	ND	-	ND	-	0.30	0.26	ND	-	ND	-
51	G	0.03	ND	-	ND	-	ND	-	ND	-	0.25	0.35	ND	-	ND	-
52	U	0.09	ND	-	ND	-	ND	-	ND	-	0.18	0.16	ND	-	ND	-
53	G	0.08	ND	-	ND	-	ND	-	ND	-	0.30	0.42	ND	-	ND	-
54	U	0.05	ND	-	ND	-	ND	-	ND	-	0.16	0.22	ND	-	ND	-
55	C	0.12	ND	-	ND	-	ND	-	ND	-	0.00	0.00	ND	-	ND	-
56	U	0.20	ND	-	ND	-	ND	-	ND	-	0.16	0.11	ND	-	ND	-
57	C	0.08	ND	-	ND	-	ND	-	ND	-	0.18	0.25	ND	-	ND	-
58	U	0.12	ND	-	ND	-	ND	-	ND	-	0.29	0.40	ND	-	ND	-

59	U	0.14	ND	-	ND	-	ND	-	ND	-	0.00	0.00	ND	-	ND	-
60	G	0.17	ND	-	ND	-	ND	-	ND	-	0.04	0.06	ND	-	ND	-
61	U	0.38	ND	-	ND	-	ND	-	ND	-	0.14	0.06	ND	-	ND	-
62	U	1.15	ND	-	ND	-	ND	-	ND	-	0.34	0.12	ND	-	ND	-
63	C	0.05	ND	-	ND	-	ND	-	ND	-	0.17	0.23	ND	-	ND	-
64	C	0.08	ND	-	ND	-	ND	-	ND	-	0.05	0.07	ND	-	ND	-
65	C	0.12	ND	-	ND	-	ND	-	ND	-	0.06	0.08	ND	-	ND	-
66	U	0.40	ND	-	ND	-	ND	-	ND	-	0.23	0.27	ND	-	ND	-
67	U	0.70	ND	-	ND	-	ND	-	ND	-	0.12	0.01	ND	-	ND	-
68	C	0.76	ND	-	ND	-	ND	-	ND	-	0.19	0.19	ND	-	ND	-
69	A	0.73	ND	-	ND	-	ND	-	ND	-	0.22	0.16	ND	-	ND	-
70	A	0.81	ND	-	ND	-	ND	-	ND	-	0.19	0.05	ND	-	ND	-
71	U	0.22	ND	-	ND	-	ND	-	ND	-	0.15	0.13	ND	-	ND	-
72	U	0.11	ND	-	ND	-	ND	-	ND	-	0.05	0.06	ND	-	ND	-
73	C	0.05	ND	-	ND	-	ND	-	ND	-	0.00	0.00	ND	-	ND	-
74	C	0.02	ND	-	ND	-	ND	-	ND	-	0.00	0.00	ND	-	ND	-
75	C	0.04	ND	-	ND	-	ND	-	ND	-	0.00	0.00	ND	-	ND	-
76	A	0.14	ND	-	ND	-	ND	-	ND	-	0.19	0.24	ND	-	ND	-
77	C	0.10	ND	-	ND	-	ND	-	ND	-	0.28	0.35	ND	-	ND	-
78	U	0.08	ND	-	ND	-	ND	-	ND	-	0.17	0.19	ND	-	ND	-
79	C	0.03	ND	-	ND	-	ND	-	ND	-	0.24	0.33	ND	-	ND	-
80	C	0.03	ND	-	ND	-	ND	-	ND	-	0.01	0.01	ND	-	ND	-
81	C	0.05	ND	-	ND	-	ND	-	ND	-	0.62	0.87	ND	-	ND	-
82	U	0.06	ND	-	ND	-	ND	-	ND	-	0.01	0.01	ND	-	ND	-
83	C	0.03	ND	-	ND	-	ND	-	ND	-	0.09	0.13	ND	-	ND	-
84	C	0.07	ND	-	ND	-	ND	-	ND	-	0.00	0.00	ND	-	ND	-
85	U	0.12	ND	-	ND	-	ND	-	ND	-	0.18	0.25	ND	-	ND	-
86	C	0.03	ND	-	ND	-	ND	-	ND	-	0.03	0.04	ND	-	ND	-
87	C	0.05	ND	-	ND	-	ND	-	ND	-	0.67	0.94	ND	-	ND	-
88	A	0.21	ND	-	ND	-	ND	-	ND	-	0.09	0.06	ND	-	ND	-
89	G	0.56	ND	-	ND	-	ND	-	ND	-	0.29	0.08	ND	-	ND	-
90	G	0.58	ND	-	ND	-	ND	-	ND	-	0.35	0.13	ND	-	ND	-

91	U	0.96	ND	-	ND	-	ND	-	ND	-	0.44	0.23	ND	-	ND	-
92	U	0.71	ND	-	ND	-	ND	-	ND	-	0.62	0.12	ND	-	ND	-
93	C	0.07	ND	-	ND	-	ND	-	ND	-	0.27	0.37	ND	-	ND	-
94	C	0.08	ND	-	ND	-	ND	-	ND	-	0.54	0.76	ND	-	ND	-
95	U	0.13	ND	-	ND	-	ND	-	ND	-	0.16	0.23	ND	-	ND	-
96	A	0.02	ND	-	ND	-	ND	-	ND	-	0.09	0.12	ND	-	ND	-
97	C	0.39	ND	-	ND	-	ND	-	ND	-	0.12	0.17	ND	-	ND	-
98	U	0.93	ND	-	ND	-	ND	-	ND	-	0.32	0.45	ND	-	ND	-
99	G	0.21	ND	-	ND	-	ND	-	ND	-	0.07	0.10	ND	-	ND	-
100	U	0.22	ND	-	ND	-	ND	-	ND	-	0.10	0.13	ND	-	ND	-
101	U	0.40	ND	-	ND	-	ND	-	ND	-	0.17	0.10	ND	-	ND	-
102	G	0.48	ND	-	ND	-	ND	-	ND	-	0.40	0.01	ND	-	ND	-
103	A	0.59	ND	-	ND	-	ND	-	ND	-	0.90	0.20	ND	-	ND	-
104	U	0.10	ND	-	ND	-	ND	-	ND	-	0.54	0.64	ND	-	ND	-
105	C	0.03	ND	-	ND	-	ND	-	ND	-	0.16	0.22	ND	-	ND	-
106	C	0.01	ND	-	ND	-	ND	-	ND	-	0.16	0.22	ND	-	ND	-
107	C	0.05	ND	-	ND	-	ND	-	0.27	-	0.64	0.91	ND	-	ND	-
108	G	0.10	ND	-	ND	-	ND	-	0.86	-	0.04	0.06	ND	-	ND	-
109	C	0.46	ND	-	ND	-	ND	-	0.39	-	0.06	0.08	ND	-	ND	-
110	G	1.47	ND	-	ND	-	ND	-	0.31	-	0.59	0.74	ND	-	ND	-
111	G	0.61	ND	-	ND	-	ND	-	0.19	-	0.60	0.85	ND	-	ND	-
112	G	0.37	ND	-	ND	-	ND	-	0.31	-	0.18	0.25	ND	-	ND	-
113	U	1.06	ND	-	ND	-	1.20	0.64	1.18	-	0.59	0.83	ND	-	ND	-
114	C	0.10	ND	-	ND	-	0.05	0.01	0.3	-	0.07	0.09	ND	-	ND	-
115	G	0.06	ND	-	ND	-	0.00	0.00	0.14	-	2.09	2.75	ND	-	ND	-
116	G	0.04	ND	-	ND	-	0.00	0.00	0	-	0.59	0.83	ND	-	ND	-
117	G	0.12	ND	-	ND	-	0.00	0.00	0	-	0.39	0.54	ND	-	ND	-
118	A	0.11	ND	-	ND	-	0.00	0.00	0.31	-	0.07	0.09	ND	-	ND	-
119	C	0.24	0.02	0.03	ND	-	0.00	0.00	5.29	-	0.77	1.08	ND	-	ND	-
120	A	0.99	0.32	0.29	ND	-	0.54	0.12	0.83	-	0.75	0.14	ND	-	ND	-
121	G	0.47	0.35	0.34	ND	-	0.55	0.25	0.32	-	0.30	0.23	ND	-	ND	-
122	U	0.87	0.45	0.38	ND	-	0.78	0.26	0.85	-	0.53	0.28	ND	-	ND	-

123	U	0.58	0.33	0.31	ND	-	0.62	0.17	1.29	-	0.39	0.23	ND	-	ND	-
124	G	0.08	0.07	0.05	ND	-	0.16	0.12	0.49	-	0.05	0.07	ND	-	ND	-
125	G	0.10	0.02	0.02	ND	-	0.06	0.10	0.44	-	0.02	0.01	ND	-	ND	-
126	C	0.10	0.02	0.03	ND	-	0.07	0.02	0.99	-	0.05	0.06	ND	-	ND	-
127	G	0.01	0.04	0.03	ND	-	0.14	0.03	0.63	-	0.19	0.04	ND	-	ND	-
128	C	0.02	0.06	0.09	ND	-	0.03	0.06	0.17	-	0.00	0.00	ND	-	ND	-
129	C	0.17	0.15	0.21	ND	-	0.06	0.10	0	-	0.00	0.00	ND	-	ND	-
130	C	0.05	0.03	0.04	ND	-	0.00	0.00	1.67	-	0.00	0.00	ND	-	ND	-
131	A	0.10	0.04	0.06	ND	-	0.03	0.06	0.5	-	0.28	0.28	ND	-	ND	-
132	A	0.21	0.09	0.10	ND	-	0.16	0.02	0.45	-	0.33	0.32	ND	-	ND	-
133	C	0.03	0.02	0.03	ND	-	0.00	0.00	0.42	-	0.08	0.11	ND	-	ND	-
134	G	0.05	0.03	0.02	ND	-	0.03	0.02	0.22	-	0.08	0.09	ND	-	ND	-
135	U	0.10	0.06	0.06	ND	-	0.07	0.09	0.43	-	0.26	0.13	ND	-	ND	-
136	G	0.06	0.02	0.03	ND	-	0.02	0.03	0.22	-	0.00	0.00	ND	-	ND	-
137	G	0.01	0.05	0.07	ND	-	0.03	0.05	0	-	0.00	0.00	ND	-	ND	-
138	G	0.08	0.06	0.08	ND	-	0.00	0.00	0	-	0.00	0.00	ND	-	ND	-
139	G	0.10	0.14	0.20	ND	-	0.06	0.11	0	-	0.00	0.00	ND	-	ND	-
140	C	0.05	0.07	0.09	ND	-	0.00	0.00	0.23	-	0.08	0.11	ND	-	ND	-
141	U	0.07	0.06	0.08	ND	-	0.01	0.01	0.29	-	0.08	0.11	ND	-	ND	-
142	G	0.05	0.03	0.02	ND	-	0.03	0.04	0	-	0.11	0.16	ND	-	ND	-
143	G	0.06	0.06	0.05	ND	-	0.00	0.00	0.43	-	0.08	0.11	ND	-	ND	-
144	A	0.08	0.08	0.11	ND	-	0.01	0.02	0.65	-	0.18	0.25	ND	-	ND	-
145	U	0.17	0.19	0.26	ND	-	0.08	0.13	2.53	-	0.07	0.09	ND	-	ND	-
146	A	0.67	0.79	0.34	ND	-	0.93	0.25	0.49	-	2.52	2.07	ND	-	ND	-
147	C	0.05	0.18	0.25	ND	-	0.11	0.16	0.26	-	0.60	0.28	ND	-	ND	-
148	G	0.07	0.01	0.01	ND	-	0.02	0.04	0.01	-	0.15	0.04	ND	-	ND	-
149	A	0.00	0	0.00	ND	-	0.00	0.00	0	-	0.10	0.14	ND	-	ND	-
150	G	0.09	0	0.00	ND	-	0.00	0.00	0	-	0.56	0.78	ND	-	ND	-
151	G	ND	0.17	0.24	ND	-	0.00	0.00	0	-	2.70	3.81	ND	-	ND	-
152	G	ND	0	0.00	ND	-	0.22	0.38	0	-	0.63	0.89	ND	-	ND	-
153	A	ND	0.1	0.10	ND	-	0.00	0.00	0	-	3.37	4.76	ND	-	ND	-
154	A	ND	0.68	0.27	ND	-	0.47	0.10	0	-	0.57	0.81	ND	-	ND	-

155	U	ND	1.24	0.41	ND	-	1.44	0.12	0.75	-	1.15	0.60	ND	-	ND	-
156	U	0.83	1.78	0.64	ND	-	2.28	0.41	0.67	-	1.21	0.82	ND	-	ND	-
157	U	0.84	1.5	0.71	ND	-	1.97	0.15	1.15	-	0.45	0.35	ND	-	ND	-
158	C	0.02	0.36	0.27	ND	-	0.30	0.15	0.73	-	0.06	0.08	ND	-	ND	-
159	G	0.10	0.09	0.06	ND	-	0.02	0.04	0.18	-	0.06	0.08	ND	-	ND	-
160	U	0.10	0.18	0.02	ND	-	0.07	0.08	0.23	-	0.06	0.08	ND	-	0.18	0.03
161	G	0.31	0.23	0.05	ND	-	0.30	0.24	0	-	0.16	0.22	ND	-	0.23	0.07
162	A	1.64	2	0.81	ND	-	2.74	0.79	0.62	-	1.15	1.36	0.62	1.03	2.00	1.00
163	G	0.10	0.15	0.02	ND	-	0.09	0.14	0.16	-	0.65	0.78	0.06	0.06	0.15	0.03
164	G	0.05	0.03	0.00	0.09	0.08	0.02	0.03	0	-	0.04	0.06	0.63	1.06	0.03	0.01
165	A	0.11	0.03	0.02	2.20	0.40	0.06	0.05	0	-	0.13	0.18	0.44	0.72	0.03	0.03
166	A	0.16	0.24	0.08	0.10	0.07	0.22	0.09	0.1	-	0.19	0.26	0.09	0.16	0.24	0.10
167	G	0.11	0.34	0.09	0.01	0.01	0.34	0.08	0.27	-	0.40	0.15	0.12	0.21	0.34	0.11
168	A	0.39	0.57	0.12	0.03	0.05	0.56	0.04	0.4	-	0.60	0.16	0.28	0.31	0.57	0.15
169	C	0.05	0.14	0.13	0.13	0.03	0.07	0.08	1.72	0.98	0.19	0.04	0.16	0.15	0.14	0.16
170	G	0.05	0.08	0.09	0.10	0.05	0.09	0.04	0	0.26	0.27	0.11	0.29	0.29	0.08	0.11
171	A	0.19	0.17	0.03	0.51	0.04	0.17	0.04	0.08	0.26	0.36	0.18	0.17	0.24	0.17	0.04
172	C	0.10	0.39	0.28	0.08	0.11	0.26	0.04	1.81	0.57	0.23	0.32	0.28	0.26	0.39	0.34
173	G	0.10	0.19	0.05	0.00	0.01	0.47	0.10	0.17	0.14	0.33	0.35	0.05	0.09	0.19	0.06
174	C	0.00	0.03	0.04	0.18	0.06	0.03	0.04	0.11	0.06	0.30	0.40	0.06	0.10	0.03	0.04
175	G	0.03	0.08	0.10	0.28	0.18	0.07	0.06	0.24	0.15	0.11	0.10	0.25	0.34	0.08	0.13
176	U	0.05	0	0.00	0.33	0.14	0.00	0.01	0.01	0.19	0.22	0.31	0.17	0.24	0.00	0.01
177	U	0.11	0.31	0.05	0.16	0.05	0.39	0.05	0.15	0.19	0.08	0.11	0.33	0.15	0.31	0.06
178	C	0.12	0.62	0.17	0.20	0.12	0.56	0.10	0.25	0.15	0.44	0.16	0.1	0.12	0.62	0.21
179	G	0.06	0.48	0.09	0.09	0.05	0.13	0.02	0	0.03	0.54	0.25	0.23	0.17	0.48	0.11
180	C	0.00	0	0.00	0.00	0.00	0.01	0.02	0.08	0.06	0.00	0.00	0.13	0.22	0.00	0.00
181	C	0.03	0.04	0.03	0.06	0.05	0.10	0.11	0.22	0.11	0.02	0.03	0.18	0.31	0.04	0.04
182	G	0.33	0.42	0.18	1.93	0.08	1.05	0.21	1.38	0.64	0.63	0.14	0.28	0.27	0.42	0.22
183	G	0.43	1.45	0.28	0.98	0.07	2.54	0.37	0.5	0.84	1.74	0.64	0.48	0.35	1.45	0.34
184	C	0.22	0.92	0.34	0.64	0.18	0.98	0.12	0.82	0.38	1.06	0.40	0.2	0.22	0.92	0.42
185	C	0.18	0.98	0.30	1.11	0.07	1.46	0.35	0.75	0.65	1.21	0.40	0.35	0.21	0.98	0.37
186	G	0.05	0.12	0.08	0.00	0.00	0.10	0.06	0	0.06	0.50	0.07	0.53	0.71	0.12	0.10

187	G	0.21	0.34	0.24	0.00	0.00	0.33	0.36	0.05	0.12	2.98	1.89	0.66	0.31	0.34	0.30
188	C	-1.00	0.85	0.60	0.00	0.00	0.39	0.49	0	0.13	1.33	1.03	1.38	0.83	0.85	0.74
189	G	0.00	0.35	0.22	0.00	0.00	0.22	0.38	0	0.06	0.31	0.43	0.3	0.24	0.35	0.27
190	A	0.04	0.54	0.26	0.00	0.00	0.10	0.12	0	0.17	0.36	0.34	0.57	0.34	0.54	0.32
191	U	0.08	0.61	0.10	**Δ**	**Δ**	0.22	0.18	**Δ**	**Δ**	0.06	0.08	0.57	0.10	0.61	0.13
192	U	0.18	2	1.51	**Δ**	**Δ**	1.24	0.36	**Δ**	**Δ**	0.00	0.00	1.01	0.91	2.00	1.85
193	A	0.11	1.26	0.06	**Δ**	**Δ**	1.53	0.40	**Δ**	**Δ**	0.36	0.01	0.57	0.31	1.26	0.07
194	A	0.10	***Δ	**Δ**	**Δ**	**Δ**	1.57	0.40	**Δ**	**Δ**	0.38	0.01	0.33	0.19	0.85	0.13
195	A	0.11	**Δ**	**Δ**	**Δ**	**Δ**	1.20	0.29	**Δ**	**Δ**	0.34	0.20	0.07	0.04	0.61	0.04
196	A	0.11	**Δ**	**Δ**	**Δ**	**Δ**	0.36	0.05	**Δ**	**Δ**	0.48	0.07	0.1	0.08	0.55	0.04
197	G	0.33	**Δ**	**Δ**	**Δ**	**Δ**	0.52	0.91	**Δ**	**Δ**	0.52	0.06	0.04	0.05	0.76	0.09
198	U	0.39	**Δ**	**Δ**	**Δ**	**Δ**	0.14	0.24	**Δ**	**Δ**	0.28	0.39	0.23	0.33	1.29	0.25
199	G	0.51	0.85	0.11	**Δ**	**Δ**	0.05	0.09	**Δ**	**Δ**	**Δ**	**Δ**	0.18	0.29	2.00	2.40
200	A	0.54	0.61	0.03	**Δ**	**Δ**	0.53	0.08	**Δ**	**Δ**	**Δ**	**Δ**	0.54	0.24	1.20	0.06
201	A	0.56	0.55	0.03	**Δ**	**Δ**	1.02	0.16	**Δ**	**Δ**	**Δ**	**Δ**	0.71	0.48	1.06	0.12
202	A	0.46	0.76	0.07	**Δ**	**Δ**	0.99	0.52	**Δ**	**Δ**	**Δ**	**Δ**	0.9	0.44	0.32	0.04
203	G	0.58	1.29	0.20	**Δ**	**Δ**	1.29	2.23	**Δ**	**Δ**	**Δ**	**Δ**	0.93	0.28	0.07	0.03
204	U	0.82	2	1.96	**Δ**	**Δ**	1.24	0.34	**Δ**	**Δ**	**Δ**	**Δ**	0.47	0.42	0.14	0.07
205	A	0.67	1.2	0.05	**Δ**	**Δ**	0.91	0.21	**Δ**	**Δ**	**Δ**	**Δ**	0.72	0.15	0.09	0.05
206	A	0.59	1.06	0.09	**Δ**	**Δ**	0.41	0.17	**Δ**	**Δ**	**Δ**	**Δ**	0.52	0.36	0.21	0.08
207	A	0.15	0.32	0.03	0.00	0.00	0.19	0.03	0	0.15	0.39	0.16	0.42	0.29	**Δ**	**Δ**
208	C	0.01	0.07	0.02	0.00	0.00	0.01	0.01	0	0.27	0.11	0.06	0.11	0.10	**Δ**	**Δ**
209	U	0.04	0.14	0.06	0.11	0.07	0.10	0.06	1.04	0.22	0.32	0.06	0.14	0.05	**Δ**	**Δ**
210	C	0.01	0.09	0.04	0.14	0.09	0.04	0.02	1.92	0.66	0.22	0.17	0.13	0.19	**Δ**	**Δ**
211	U	0.03	0.21	0.06	0.18	0.04	0.16	0.16	1.75	0.62	0.19	0.04	0.13	0.08	**Δ**	**Δ**
212	C	0.08	0.05	0.01	0.08	0.08	0.02	0.02	1.96	0.90	0.24	0.08	0.15	0.23	**Δ**	**Δ**
213	U	0.20	0.27	0.02	0.34	0.05	0.35	0.03	0.68	0.28	0.73	0.20	0.08	0.09	0.05	0.02

214	U	0.22	0.41	0.06	0.44	0.05	0.49	0.05	0.52	0.16	0.78	0.13	0.34	0.17	0.27	0.03
215	G	0.15	0.33	0.07	0.17	0.03	0.29	0.24	0.33	0.06	0.47	0.06	0.58	0.19	0.41	0.07
216	G	0.04	0.22	0.07	0.09	0.03	0.44	0.23	0	0.02	0.08	0.04	0.26	0.45	0.33	0.09
217	C	0.05	0.06	0.04	0.01	0.01	0.01	0.02	0.61	0.33	0.03	0.04	0.02	0.03	0.22	0.08
218	C	0.03	0.02	0.03	0.01	0.02	0.03	0.02	0.12	0.06	0.00	0.00	0.06	0.08	0.06	0.06
219	G	0.04	0.07	0.02	0.02	0.01	0.09	0.04	0	0.11	0.16	0.19	0.02	0.02	0.02	0.04
220	C	0.08	0.11	0.02	0.01	0.01	0.08	0.06	0.43	0.20	0.09	0.00	0.03	0.05	0.07	0.03
221	C	0.00	0.01	0.01	0.00	0.00	0.05	0.05	0.05	0.04	0.03	0.04	0.03	0.03	0.11	0.03
222	G	0.01	0.03	0.02	0.00	0.00	0.06	0.06	0.02	0.08	0.11	0.06	0.02	0.03	0.01	0.01
223	C	0.02	0.02	0.02	0.03	0.06	0.02	0.03	0	0.07	0.05	0.07	0.01	0.02	0.03	0.03
224	G	0.02	0	0.00	0.00	0.01	0.02	0.02	0	0.02	0.14	0.19	0	0.00	0.02	0.03
225	G	0.22	0.03	0.05	0.02	0.03	0.10	0.10	0	0.00	0.22	0.18	0	0.01	0.00	0.00
226	G	0.68	0.84	0.34	0.72	0.39	1.32	0.22	0	0.27	0.96	0.27	0.5	0.45	0.03	0.06
227	A	1.49	2	0.38	2.14	0.29	3.30	0.83	0.66	0.42	1.78	0.36	1.43	0.79	0.84	0.41
228	A	0.87	1.93	0.39	2.09	0.18	2.54	0.67	0.6	0.43	1.58	0.33	1.78	0.27	2.00	0.47
229	C	0.28	0.59	0.20	0.47	0.46	0.81	0.40	0.16	0.20	0.18	0.25	0.74	0.79	1.93	0.48
230	C	0.56	0.46	0.35	0.69	0.15	0.66	0.34	0.23	0.16	0.08	0.11	0.35	0.13	0.59	0.24
231	U	1.08	1.72	0.50	2.06	0.13	2.22	0.66	0.61	0.54	1.16	0.15	1.22	0.70	0.46	0.43
232	G	0.42	1.75	0.36	2.02	0.17	2.33	0.58	0.27	0.61	1.00	0.41	1.23	0.54	1.72	0.62
233	C	0.12	0.05	0.04	0.04	0.04	0.02	0.03	0.7	0.39	0.09	0.12	0.32	0.56	1.75	0.44
234	C	0.01	0	0.00	0.00	0.00	0.00	0.00	0.09	0.05	0.01	0.01	0.03	0.05	0.05	0.05
235	G	0.01	0.02	0.02	0.00	0.00	0.03	0.04	0	0.08	0.17	0.18	0.03	0.06	0.00	0.00
236	C	0.01	0	0.00	0.00	0.00	0.00	0.00	0.51	0.27	0.12	0.12	0.22	0.22	0.02	0.03
237	G	0.08	0.04	0.04	0.04	0.08	0.03	0.02	0.17	0.11	0.34	0.04	0.5	0.46	0.00	0.00
238	U	0.13	0.24	0.07	0.22	0.10	0.26	0.08	0.23	0.10	0.42	0.12	0.48	0.42	0.04	0.05
239	U	0.09	0.19	0.00	0.12	0.04	0.19	0.02	0.39	0.05	0.54	0.13	0.68	0.60	0.24	0.09
240	G	0.04	0.1	0.02	0.02	0.02	0.08	0.06	0	0.10	0.38	0.05	0.36	0.20	0.19	0.01
241	G	0.16	0.27	0.03	0.19	0.02	0.19	0.07	0.32	0.05	0.58	0.07	0.61	0.32	0.10	0.03
242	A	0.68	2	0.20	2.01	1.51	2.53	0.63	2.21	1.34	4.04	0.45	2.48	1.46	0.27	0.04
243	C	0.04	0.09	0.03	0.56	0.96	0.03	0.01	0.94	0.44	0.15	0.18	0.7	0.58	2.00	0.24
244	C	0.05	0.04	0.04	0.02	0.04	0.02	0.02	0.06	0.03	0.00	0.00	0.22	0.32	0.09	0.04
245	U	0.11	0.13	0.06	0.11	0.08	0.12	0.06	0.17	0.01	0.31	0.04	0.3	0.30	0.04	0.05

246	G	0.04	0.17	0.04	0.20	0.08	0.07	0.05	Δ	Δ	Δ	Δ	Δ	Δ	0.13	0.07
247	A	0.07	0.18	0.03	0.34	0.09	0.18	0.06	Δ	Δ	Δ	Δ	Δ	Δ	0.17	0.06
248	A	0.09	0.17	0.02	0.28	0.06	0.28	0.09	Δ	Δ	Δ	Δ	Δ	Δ	0.18	0.04
249	A	0.03	0.11	0.07	0.18	0.05	0.38	0.13	Δ	Δ	Δ	Δ	Δ	Δ	0.17	0.03
250	G	0.17	0.28	0.19	0.24	0.03	0.11	0.04	Δ	Δ	Δ	Δ	Δ	Δ	0.11	0.08
251	U	0.38	2	3.21	0.63	0.31	0.14	0.25	Δ	Δ	Δ	Δ	Δ	Δ	0.28	0.23
252	A	0.60	0.59	0.11	0.95	0.10	0.18	0.07	Δ	Δ	Δ	Δ	Δ	Δ	2.00	3.93
253	A	0.32	0.45	0.04	0.68	0.06	0.29	0.10	Δ	Δ	Δ	Δ	Δ	Δ	0.59	0.14
254	G	0.48	0.51	0.03	0.51	0.14	0.24	0.16	0.19	0.17	0.41	0.11	1.14	1.78	0.45	0.06
255	U	0.53	0.9	0.36	0.83	0.22	0.17	0.10	1.65	0.46	0.77	0.48	0.31	0.23	0.51	0.04
256	G	0.39	0.64	0.05	0.46	0.09	0.16	0.16	0.29	0.18	0.45	0.06	0.12	0.11	0.90	0.44
257	U	0.44	0.6	0.06	0.90	0.29	0.32	0.05	0.61	0.27	0.67	0.07	0.56	0.49	0.64	0.07
258	U	0.22	0.52	0.01	0.64	0.08	0.47	0.01	0.49	0.19	0.44	0.11	0.29	0.15	0.60	0.07
259	G	0.01	0.08	0.02	0.08	0.02	0.11	0.14	0.05	0.05	0.14	0.13	0.12	0.07	0.52	0.01
260	C	0.06	0.04	0.04	0.03	0.05	0.04	0.07	0.87	0.34	0.14	0.08	0.34	0.30	0.08	0.02
261	G	0.15	0.21	0.05	0.38	0.11	0.50	0.22	0.25	0.21	0.21	0.04	0.16	0.11	0.04	0.06
262	C	0.13	0.1	0.08	0.08	0.11	0.17	0.17	0.37	0.11	0.09	0.12	0.38	0.65	0.21	0.07
263	U	0.17	0.21	0.03	0.17	0.12	0.14	0.14	0.22	0.16	0.39	0.01	0.19	0.20	0.10	0.10
264	C	0.11	0.17	0.24	0.10	0.12	0.12	0.18	1.57	0.67	0.24	0.05	0.7	0.93	0.21	0.04
265	G	0.35	0.22	0.09	0.44	0.07	0.42	0.11	0.17	0.19	0.45	0.03	0.43	0.37	0.17	0.29
266	G	0.42	0.55	0.06	0.41	0.12	0.69	0.13	0.26	0.13	0.80	0.12	0.45	0.30	0.22	0.11
267	A	0.82	1.05	0.07	1.52	0.07	1.23	0.18	2.03	0.51	0.63	0.22	0.88	0.62	0.55	0.07
268	U	0.66	2	1.20	1.11	0.45	0.99	0.59	5.68	2.86	1.48	2.09	1.9	2.45	1.05	0.09
269	A	0.41	0.57	0.19	0.84	0.22	0.73	0.26	0.29	0.12	0.59	0.11	0.42	0.30	2.00	1.47
270	U	0.40	0.55	0.15	0.77	0.18	0.68	0.28	0.24	0.14	0.74	0.03	0.37	0.23	0.57	0.24
271	G	0.04	0.03	0.02	0.02	0.02	0.05	0.09	0.00	0.03	0.16	0.07	0.16	0.11	0.55	0.19
272	G	0.02	0	0.00	0.00	0.00	0.00	0.00	0.00	0.00	0.00	0.00	0.32	0.55	0.03	0.03
273	G	0.02	0	0.00	0.00	0.00	0.00	0.00	0.00	0.00	0.00	0.00	0.26	0.44	0.00	0.00
274	G	0.01	0	0.00	0.00	0.00	0.00	0.00	0.00	0.00	0.00	0.00	0.53	0.91	0.00	0.00
275	C	0.06	0	0.00	0.00	0.00	0.00	0.00	1.37	0.79	0.00	0.00	0.53	0.88	0.00	0.00

276	A	0.13	0.02	0.02	0.01	0.01	0.08	0.07	0.00	0.06	0.21	0.29	0.26	0.45	0.00	0.00
277	A	0.16	0.08	0.06	0.08	0.03	0.13	0.10	0.00	0.07	0.38	0.28	0.23	0.34	0.02	0.03
278	G	0.18	0.17	0.09	0.11	0.04	0.19	0.23	0.00	0.09	0.64	0.31	0.15	0.14	0.08	0.07
279	A	0.40	0.43	0.08	0.55	0.07	0.52	0.26	0.17	0.14	0.90	0.54	0.29	0.33	0.17	0.11
280	A	0.32	0.6	0.06	0.72	0.06	0.67	0.18	0.34	0.14	0.81	0.34	0.34	0.32	0.43	0.09
281	U	0.49	0.82	0.10	1.14	0.13	0.90	0.11	0.70	0.07	0.93	0.38	0.47	0.42	0.60	0.08
282	U	0.20	0.36	0.20	0.56	0.15	0.20	0.12	1.86	0.94	0.08	0.11	0.32	0.30	0.82	0.12
283	A	0.09	0.05	0.04	0.08	0.03	0.07	0.07	0.00	0.02	0.05	0.06	0	0.00	0.36	0.25
284	A	0.03	0.03	0.01	0.02	0.02	0.04	0.05	0.03	0.02	0.05	0.01	0.08	0.12	0.05	0.05
285	G	0.01	0.01	0.00	0.03	0.04	0.02	0.03	0.06	0.03	0.03	0.04	0.1	0.18	0.03	0.02
286	C	0.09	0.09	0.07	0.06	0.04	0.10	0.08	0.51	0.28	0.01	0.01	0.14	0.16	0.01	0.01
287	C	0.09	0.04	0.06	0.07	0.06	0.05	0.09	0.62	0.32	0.03	0.04	0.35	0.32	0.09	0.08
288	A	0.08	0.05	0.04	0.07	0.07	0.12	0.06	0.04	0.04	0.07	0.05	0.25	0.31	0.04	0.07
289	G	0.02	0.01	0.01	0.05	0.08	0.00	0.00	0.87	0.48	0.00	0.00	0.22	0.38	0.05	0.05
290	C	0.13	0.41	0.29	0.37	0.48	0.17	0.30	3.56	1.90	0.90	1.27	0.73	0.90	0.01	0.02
291	A	0.71	0.8	0.15	0.62	0.44	0.94	0.34	0.10	0.46	0.87	0.47	0.39	0.38	0.41	0.36
292	U	0.48	1.11	0.38	0.76	0.34	1.07	0.10	0.14	0.46	1.33	0.40	0.63	0.53	0.80	0.18
293	G	0.34	0.64	0.15	0.60	0.36	0.65	0.13	0.09	0.26	0.75	0.11	0.24	0.20	1.11	0.46
294	A	0.21	0.47	0.05	0.38	0.15	0.42	0.08	0.12	0.18	0.49	0.23	0.29	0.01	0.64	0.19
295	A	0.19	0.37	0.06	0.34	0.03	0.30	0.04	0.18	0.08	0.37	0.07	0.21	0.05	0.47	0.07
296	C	0.14	0.27	0.08	0.29	0.12	0.25	0.01	0.32	0.05	0.31	0.04	0.17	0.13	0.37	0.07
297	G	0.17	0.15	0.07	0.25	0.16	0.11	0.05	0.06	0.06	0.18	0.12	0.07	0.06	0.27	0.10
298	U	0.26	0.33	0.10	0.25	0.08	0.27	0.06	0.40	0.20	0.00	0.00	0.13	0.13	0.15	0.08
299	U	0.34	1.01	1.04	0.64	0.71	0.24	0.06	2.71	1.52	0.62	0.88	0.12	0.08	0.33	0.12
300	A	1.04	1.09	0.07	1.11	0.75	1.19	0.37	0.72	0.13	1.31	0.32	0.9	0.71	1.01	1.28
301	U	0.41	0.77	0.14	1.12	0.72	0.86	0.33	0.29	0.12	0.67	0.21	0.61	0.16	1.09	0.09
302	G	0.79	1.45	0.07	1.37	0.65	1.45	0.54	1.03	0.11	1.20	0.03	0.91	0.69	0.77	0.18
303	U	1.29	1.87	1.14	1.68	0.22	0.98	0.34	3.15	1.60	0.53	0.75	1.13	0.63	1.45	0.08
304	A	1.80	1.39	0.19	1.82	0.19	1.58	0.52	0.67	0.47	1.47	0.34	1.46	1.04	1.87	1.40
305	G	0.66	1.71	0.43	1.36	0.72	1.89	0.57	0.56	0.61	1.69	0.71	1.35	0.88	1.39	0.23
306	A	0.42	0.37	0.05	0.57	0.31	0.48	0.05	0.22	0.16	0.44	0.27	0.3	0.16	1.71	0.53
307	A	0.36	0.12	0.09	0.16	0.06	0.13	0.12	0.50	0.15	0.23	0.33	0.05	0.08	0.37	0.06

308	C	0.33	0.38	0.50	0.43	0.22	0.10	0.17	6.59	3.64	0.00	0.00	0.36	0.53	0.12	0.12
309	A	0.62	0.26	0.05	0.41	0.19	0.29	0.11	0.24	0.10	0.34	0.27	0.34	0.09	0.38	0.61
310	A	0.20	0.55	0.09	0.48	0.40	0.56	0.05	0.15	0.24	0.60	0.25	0.46	0.08	0.26	0.06
311	U	0.32	0.66	0.06	0.79	0.36	0.70	0.10	0.21	0.30	0.69	0.23	1.05	0.71	0.55	0.11
312	U	0.79	0.62	0.08	0.88	0.07	0.66	0.06	0.29	0.23	0.74	0.25	0.69	0.18	0.66	0.08
313	G	0.59	0.47	0.05	0.95	0.26	0.34	0.10	0.25	0.17	0.58	0.30	0.8	0.90	0.62	0.10
314	A	0.72	0.51	0.07	0.80	0.21	0.55	0.09	0.25	0.23	0.62	0.37	1.13	0.77	0.47	0.06
315	A	0.23	0.54	0.13	0.59	0.42	0.61	0.05	0.29	0.15	0.61	0.23	1.31	1.23	0.51	0.09
316	G	0.01	0.01	0.01	0.24	0.31	0.00	0.00	0.35	0.20	0.01	0.01	0.59	1.00	0.54	0.16
317	C	0.15	0.25	0.36	0.22	0.15	0.13	0.23	4.64	2.46	0.29	0.40	0.97	1.31	0.01	0.02
318	A	0.28	0.22	0.03	0.11	0.16	0.20	0.11	0.10	0.14	0.32	0.45	0.07	0.08	0.25	0.44
319	G	0.01	0	0.00	0.00	0.01	0.00	0.00	0.00	0.00	0.00	0.00	0.09	0.16	0.22	0.04
320	G	0.02	0	0.00	0.11	0.23	0.00	0.00	0.00	0.00	0.00	0.00	0.18	0.31	0.00	0.00
321	C	0.25	0.01	0.01	0.31	0.40	0.00	0.00	0.00	0.00	0.00	0.00	0.18	0.31	0.00	0.00
322	U	0.42	0.37	0.11	0.26	0.23	0.38	0.21	0.17	0.05	0.22	0.30	0.37	0.44	0.01	0.02
323	U	0.40	0.54	0.13	0.49	0.69	0.57	0.10	0.74	0.22	0.44	0.04	0.53	0.43	0.37	0.13
324	U	0.73	1.35	0.92	0.81	0.69	0.25	0.25	4.45	2.36	0.30	0.42	0.4	0.27	0.54	0.16
325	A	0.89	0.98	0.14	1.14	0.44	0.88	0.03	0.87	0.07	0.91	0.16	0.92	0.20	1.35	1.12
326	A	0.85	0.69	0.12	0.95	0.20	0.64	0.04	0.50	0.19	0.68	0.29	0.71	0.16	0.98	0.17
327	A	0.46	0.55	0.09	0.66	0.42	0.55	0.05	0.38	0.12	0.62	0.30	0.47	0.42	0.69	0.14
328	G	0.08	0.03	0.04	0.26	0.31	0.00	0.01	0.04	0.04	0.03	0.04	0.06	0.07	0.55	0.11
329	A	0.13	0.03	0.04	0.05	0.05	0.02	0.03	0.63	0.33	0.06	0.08	0.11	0.19	0.03	0.05
330	C	0.07	0.08	0.11	0.16	0.15	0.04	0.07	6.13	3.51	0.02	0.03	0.67	1.16	0.03	0.05
331	A	0.09	0.01	0.01	0.03	0.02	0.07	0.04	0.01	0.01	0.01	0.01	0.02	0.03	0.08	0.14
332	C	0.01	0	0.00	0.01	0.01	0.02	0.02	0.05	0.03	0.01	0.01	0.02	0.03	0.01	0.01
333	G	0.00	0	0.00	0.05	0.03	0.04	0.04	0.00	0.00	0.01	0.01	0.17	0.27	0.00	0.01
334	G	0.03	0.03	0.04	0.05	0.08	0.05	0.09	0.00	0.00	0.04	0.05	0.23	0.30	0.00	0.00
335	G	0.01	0	0.00	0.07	0.03	0.03	0.05	0.00	0.00	0.01	0.01	0.22	0.38	0.03	0.04
336	G	0.11	0.02	0.03	0.07	0.08	0.06	0.10	0.07	0.04	0.08	0.11	0.34	0.54	0.00	0.01
337	A	0.08	0.14	0.04	0.09	0.08	0.18	0.14	0.39	0.13	0.42	0.23	0.37	0.32	0.02	0.03
338	G	0.10	0.17	0.03	0.18	0.16	0.16	0.17	0.74	0.38	0.19	0.27	0.38	0.35	0.14	0.05
339	U	0.34	0.26	0.37	0.32	0.33	0.20	0.34	6.23	3.45	0.88	1.24	0.68	0.85	0.17	0.04

340	A	0.20	0.39	0.07	0.67	0.53	0.40	0.18	0.29	0.04	0.57	0.17	0.26	0.23	0.26	0.45
341	A	0.12	0.29	0.07	0.34	0.28	0.28	0.15	0.09	0.08	0.40	0.09	0.15	0.13	0.39	0.09
342	A	0.17	0.35	0.13	0.23	0.13	0.23	0.20	0.07	0.12	0.37	0.21	0.13	0.10	0.29	0.08
343	G	0.05	0.07	0.07	0.26	0.23	0.00	0.00	0.00	0.00	0.04	0.05	0.02	0.03	0.35	0.16
344	G	0.08	0.1	0.07	0.13	0.16	0.00	0.00	0.08	0.05	0.00	0.00	0.03	0.03	0.07	0.09
345	U	0.34	0.39	0.20	0.21	0.26	0.39	0.29	0.82	0.39	0.09	0.12	0.23	0.20	0.10	0.08
346	U	0.53	0.96	0.31	0.44	0.29	0.39	0.31	6.02	3.32	0.77	1.08	0.61	0.56	0.39	0.25
347	A	0.48	0.83	0.19	0.74	0.27	0.71	0.09	0.96	0.26	0.92	0.20	0.37	0.26	0.96	0.38
348	A	0.29	0.8	0.19	0.54	0.15	0.68	0.08	0.85	0.23	0.82	0.23	0.3	0.26	0.83	0.24
349	A	0.38	0.85	0.23	0.57	0.14	0.70	0.09	1.28	0.53	0.59	0.83	0.3	0.28	0.80	0.24
350	U	0.43	1.63	1.51	0.59	0.18	0.37	0.33	4.84	2.67	1.97	2.79	0.43	0.33	0.85	0.28
351	A	0.31	0.68	0.06	0.66	0.21	0.74	0.11	0.26	0.12	0.48	0.18	0.27	0.15	1.63	1.85
352	U	0.27	0.71	0.04	0.48	0.30	0.84	0.18	0.25	0.16	0.61	0.08	0.51	0.05	0.68	0.08
353	G	0.24	0.62	0.11	0.80	0.35	0.70	0.24	0.15	0.28	0.48	0.08	0.56	0.19	0.71	0.05
354	C	0.19	0.1	0.14	0.51	0.25	0.05	0.09	0.48	0.16	0.00	0.00	0.13	0.08	0.62	0.14
355	U	0.33	0.22	0.16	0.16	0.18	0.19	0.16	0.13	0.10	0.10	0.14	0.1	0.07	0.10	0.17
356	G	0.26	0.33	0.03	0.34	0.26	0.24	0.12	0.02	0.20	0.21	0.14	0.07	0.06	0.22	0.19
357	A	0.39	0.47	0.28	0.13	0.17	0.60	0.39	0.02	0.48	0.45	0.45	0.18	0.16	0.33	0.04
358	U	0.03	0.1	0.09	0.07	0.08	0.08	0.07	0.22	0.10	0.05	0.07	0.1	0.16	0.47	0.34
359	C	0.03	0	0.00	0.15	0.26	0.00	0.00	0.17	0.10	0.00	0.00	0.28	0.49	0.10	0.11
360	U	0.32	0.25	0.19	0.11	0.10	0.24	0.21	0.00	0.10	0.04	0.05	0.23	0.24	0.00	0.00
361	U	0.27	0.3	0.08	0.13	0.14	0.35	0.15	0.03	0.17	0.21	0.04	0.23	0.11	0.25	0.23
362	U	0.29	0.39	0.05	0.30	0.11	0.38	0.08	0.10	0.17	0.35	0.06	0.25	0.06	0.30	0.10
363	U	0.33	0.26	0.15	0.49	0.18	0.22	0.19	0.17	0.27	0.17	0.23	0.29	0.03	0.39	0.06
364	G	0.35	0.33	0.12	0.71	0.33	0.14	0.23	0.04	0.29	0.15	0.21	0.24	0.05	0.26	0.19
365	A	0.54	0.47	0.12	0.44	0.29	0.34	0.19	0.03	0.26	0.22	0.25	0.37	0.06	0.33	0.14
366	A	0.13	0.11	0.08	0.29	0.23	0.11	0.10	0.00	0.10	0.02	0.03	0.24	0.26	0.47	0.15
367	A	0.04	0.04	0.05	0.12	0.08	0.02	0.02	0.00	0.03	0.00	0.00	0.01	0.00	0.11	0.10
368	U	0.11	0.09	0.07	0.13	0.21	0.04	0.04	0.00	0.08	0.05	0.07	0.1	0.04	0.04	0.06
369	U	0.15	0.25	0.21	0.26	0.24	0.06	0.10	0.00	0.24	0.19	0.27	0.32	0.27	0.09	0.09
370	U	0.07	0.13	0.09	0.28	0.30	0.26	0.06	0.08	0.16	0.12	0.16	0.26	0.17	0.25	0.26
371	U	0.05	0.2	0.03	0.24	0.11	0.35	0.23	0.07	0.13	0.11	0.16	0.31	0.04	0.13	0.11

372	U	0.15	0.12	0.10	0.36	0.23	0.20	0.15	0.05	0.10	0.11	0.16	0.2	0.12	0.20	0.03
373	U	0.23	0.11	0.08	0.44	0.20	0.23	0.22	0.34	0.20	0.16	0.22	0.29	0.25	0.12	0.13
374	G	0.10	0.06	0.06	0.54	0.22	0.01	0.02	0.05	0.06	0.00	0.00	0.14	0.11	0.11	0.10
375	A	0.23	0.14	0.09	0.09	0.13	0.18	0.25	0.05	0.08	0.00	0.00	0.21	0.09	0.06	0.08
376	U	0.14	0.16	0.11	0.12	0.08	0.22	0.17	0.10	0.10	0.02	0.03	0.21	0.11	0.14	0.12
377	U	0.08	0.06	0.07	0.12	0.12	0.11	0.10	0.07	0.03	0.02	0.02	0.17	0.22	0.16	0.14
378	U	0.15	0.05	0.05	0.09	0.08	0.07	0.11	0.15	0.08	0.01	0.01	0.14	0.12	0.06	0.09
379	U	0.06	0.05	0.05	0.12	0.04	0.05	0.08	0.26	0.13	0.06	0.05	0.22	0.18	0.05	0.06
380	G	0.02	0.07	0.07	0.19	0.07	0.00	0.00	0.04	0.06	0.05	0.06	0.08	0.06	0.05	0.06
381	U	0.15	0.02	0.02	0.13	0.12	0.06	0.10	0.31	0.11	0.01	0.01	0.12	0.09	0.07	0.09
382	G	0.06	0.04	0.06	0.21	0.12	0.00	0.00	0.00	0.05	0.00	0.00	0.06	0.07	0.02	0.03
383	A	0.07	0.03	0.04	0.10	0.12	0.05	0.04	0.00	0.06	0.00	0.00	0.13	0.16	0.04	0.08
384	A	0.08	0.12	0.06	0.13	0.17	0.10	0.06	0.02	0.10	0.05	0.06	0.18	0.16	0.03	0.05
385	G	0.19	0.07	0.04	0.28	0.35	0.00	0.00	0.01	0.11	0.06	0.07	0.12	0.17	0.12	0.07
386	G	0.28	0.21	0.01	0.65	0.92	0.00	0.00	0.15	0.12	0.17	0.11	0.28	0.07	0.07	0.04
387	A	0.83	1.03	0.53	0.59	0.56	1.25	0.75	0.56	0.51	0.39	0.54	0.67	0.11	0.21	0.01
388	U	0.77	1.17	0.58	ND	-	0.19	0.28	2.52	1.31	0.45	0.64	0.34	0.23	1.03	0.65

* RD: Mean reactivity data for triplicate experiments

** Standard deviation

***Δ Deleted nucleotides in the relevant clones
